# Consumer Safety and Pesticide Residues: Evaluating Mitigation Protocols for Greengrocery

**DOI:** 10.3390/jox14040088

**Published:** 2024-11-01

**Authors:** Diana Ionela Popescu (Stegarus), Corina Mihaela Oprita (Cioara), Radu Tamaian, Violeta-Carolina Niculescu

**Affiliations:** 1 National Research and Development Institute for Cryogenic and Isotopic Technologies—ICSI Ramnicu Valcea, 4th Uzinei Street, P.O. Box Raureni 7, 240050 Ramnicu Valcea, Romania; diana.stegarus@icsi.ro (D.I.P.);; 2 Doctoral School of Applied Sciences, Ovidius University Constanta, 124 Mamaia Blvd, 1st University Alley, 900470 Constanta, Romania

**Keywords:** agriculture, human safety, fruits, mitigation procedures, pesticides, risk assessment, vegetables

## Abstract

The application of pesticides remains a necessary measure for pest management in agriculture, particularly in the cultivation of fruits and vegetables. After harvest, the presence of pesticide residues in greengrocery (fruits and vegetables) is significantly influenced by various factors, including storage conditions, handling practices, and subsequent processing methods. The mitigation of these residues to levels compliant with regulated maximum thresholds ensures the safety of raw and processed fruits and vegetables for consumption. A contemporary survey of pesticide residues in greengrocery has gathered considerable attention from consumers, driven by concerns over the potential health risk of pesticide exposure. Consequently, consumers want to be extensively informed about household processing techniques to minimize associated risks. Meanwhile, a critical question arises: does household processing effectively eliminate pesticide residues? A comprehensive review of the literature reveals that conventional methods, such as washing and soaking, offer only limited reduction in residue levels, while emerging treatments, suitable both at household and industrial scale, demonstrate increased efficiency in residues mitigation. This study aims to emphasise the ubiquitous use of pesticides in crop cultivation while providing recommendations for the implementation of efficient treatment protocols to address residue concerns. Following upon available evidence and database mining, the worldwide purpose must be to outline agriculturally and economically viable strategies that prioritize both the health and safety of consumers, as well as the green cultivation and processing of fruits and vegetables.

## 1. Introduction

Pesticides represent compounds used for killing, repelling, or controlling forms of animal and plant life considered dangerous for agriculture or domestic life [1]. This generic term includes several chemical classes: herbicides (used to destroy or control weeds or various undesired vegetation); insecticides (used to kill and control insects); and fungicides (used to control fungi, and also playing a role in protecting crops) [1].

The Food and Agriculture Organization of the United Nations (FAO) defined a pesticide as “any substance or mixture of substances or biological ingredients intended for repelling, destroying or controlling any pest or regulating plant growth” [2].

The pesticide usage in Europe did not decrease despite the intensive debates regarding the sustainability of agriculture and the appearance of new pesticides that can be applied at low dosage [3]. Approximately 350,000 tons per year were sold between 2011 and 2020 in the European Union, the majority being applied in agriculture [3]. The EU provided European Commission-EC Regulation 1107/2009 for the commercialisation of plant protection compounds, including pesticides [4]. Furthermore, EC Regulation 396/2005 imposed a harmonized Maximum Residue Level (MRL) for all agriculture products containing pesticides [5].

Due to the intensive use, humans and animals are exposed to pesticide residues through various sources at work, school, their homes, or other places. Pesticides can be ingested by eating, drinking, and breathing, but can also get inside the body through skin [3].

The issue of food contamination with residual pesticides is considered an emerging problem. Recently, agricultural products have been obtained using procedures that rely on the intensive application of pesticides [6]. Despite scientific efforts, “ecological agriculture” cannot supply the required food quantity for the entire world population [7]. Considering that around 80–85% of residual pesticides enter the human body from food, special attention must be given to the agriculture and food industry to provide high-quality products [8,9]. Not all countries can provide their population with enough agricultural raw materials or products resulting from their processing, and the quality of imported food also represents an important issue [6].

In order to reduce the health risk caused by the residual amounts of pesticides, it is mandatory to validate information regarding the level of contamination [7].

Many pesticides are used, including combinations of two, three, or more active compounds with various compositions [10].

All of the above-mentioned factors, together with the geographical location, soil, and climate are influencing the residual amounts of pesticides within the final agricultural products [8,11]. The regulations and science of the residual pesticides must target every sector, from the soil to the final product, in other words, from the farm to the consumer.

Attempts to replace pesticides with alternatives have been achieved in several countries with the support of the government. Nevertheless, it was difficult to maintain the programs due to resistance from farmers that want to obtain a good harvest and quality products [12].

The use of alternatives can also be complicated by climate changes or pandemics. Furthermore, the corporations producing pesticides may be reticent to participate in the short- to medium-term business incentives to change their product portfolio [12]. Therefore, to motivate the use of alternatives, further regulations, enforcement in both developed and developing countries, and leadership by the EU are essential. Monitoring the legal trade in pesticides is considered a challenge for the governments of the countries.

The aim of this overview is to highlight the fate of pesticides from soil to final products, such as the fruits and vegetables, but also some solutions to mitigate the residual amounts of pesticides.

## 2. Classification of Pesticides, Restrictions, and MRLs

**Figure 1:**
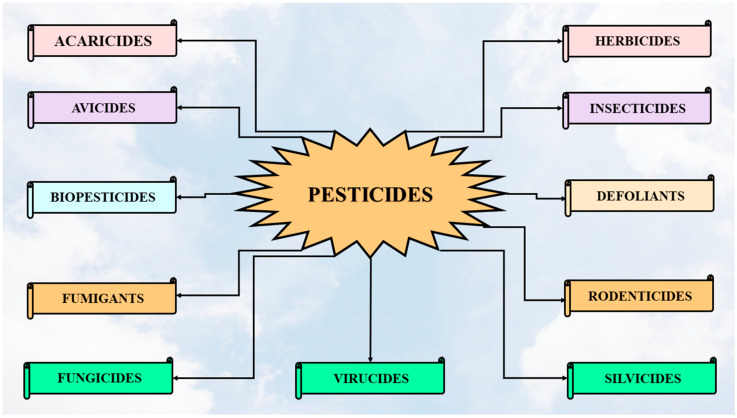
Pesticide classes according to their target and type.

Pesticides can be classified based on the target pest object and they have received special names that reflect their action. The category name of a pesticide is composed from the name of the target pest followed by the Latin word “*cide*” (killer) [13]. Figure 1 and Table 1 present the pesticides organized based on target pests [13,14].

**Figure 2:**
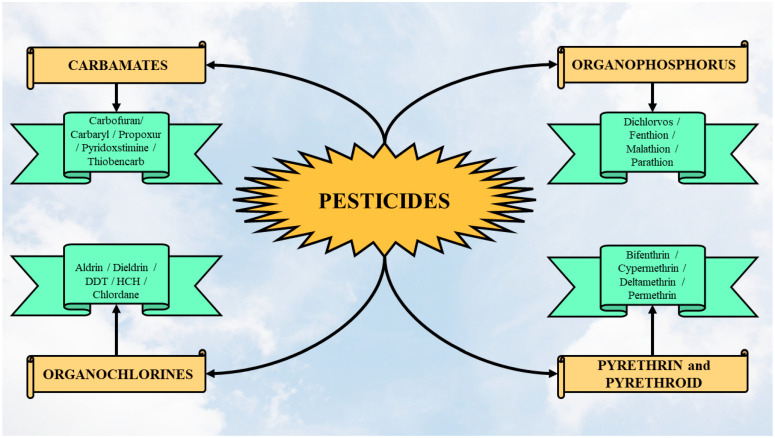
Pesticide classification based on the chemical composition.

Taking into consideration the chemical composition, pesticides can be classified into four classes: carbamates, organochlorines, organophosphorus, pyrethrins, and pyrethroids (Figure 2) [26]. The toxicity of carbamates is dependent on their molecular structure [27]. Organochlorines are classified into five subclasses (Figure 2) [28].

Carbamates are organic esters derived from N, N-dimethyl carbamic acid and they are used as herbicides, insecticides, or fungicides. The organophosphorus pesticides represent esters originating from phosphoric acid [29]. Pyrethroids are natural insecticides resulting from pyrethrum extracts of chrysanthemum flowers [29]. Synthetic pyrethroids can also be obtained by duplicating the natural pyrethrins structure, and these include cypermethrin and permethrin [30].

Maximum residue limits (MRLs) (expressed in mg/kg) are the highest levels of pesticides residues allowed to be found in food [31]. Generally, these limits were established and recommended by the World Health Organization (WHO) and the Food and Agriculture Organization (FAO). They are also imposed by specific laws in most countries, such as those of the European Commission (EC) from Europe, or the Food and Drug Administration (FDA) from the United States (US).

For exemplification, Table 2 presents a comparison of some MRLs for pesticides found in common fruits and vegetables (such as apples, grapes, potatoes, or tomatoes), according to the EC [32] and the FDA [33]. It was observed that some of the pesticides found in greengrocery are banned or under different types of restrictions, both within the European Union (EU) and the US. However, some pesticides that are not under restrictions in the EU can be found banned or under restricted use in US, such examples being propiconazole, propoxur, or captan (Table 2).

It can be noted that, overall, MRLs set by the EC were lower. For example, the MRL for malathion is 400 times higher in North America than in Europe for all selected fruits and vegetables. However, among the given examples, the case of glyphosate, which has lower imposed MRLs in the US than in Europe for grapes and potatoes, can be highlighted. Some differences have been observed by comparing MRLs for aldrin and dieldrin. If the EC recommends a combined limit (aldrin and dieldrin combined expressed as dieldrin), the US has separate MRLs for the two pesticides. Another special case is that of lindane, which has a half-life of around two weeks in soil and water. Moreover, lindane does not highly bioaccumulate in plants, but its isomers are lipophilic, so they can accumulate in the fatty tissues of animals. Therefore, the general exposure of humans to lindane is through the diet, despite its continuously decreasing levels in environmental samples [37].

To be able to identify the occurrence and risks of all banned or restricted pesticide residues in fruits and vegetables, studies on the following must be completed: (1) analyse thoroughly the banned pesticides levels in various greengrocery items based on the results of a multiannual international survey; (2) assess the human health risks from human dietary exposure to banned pesticides; and (3) fulfil an initial classification involving matrix-ranking subclasses and pesticide types [38]. All of these results will provide adequate data for the estimation of contamination with banned pesticides in greengrocery.

## 3. Consumption of Pesticides and Soil Detection Strategies

**Figure 3:**
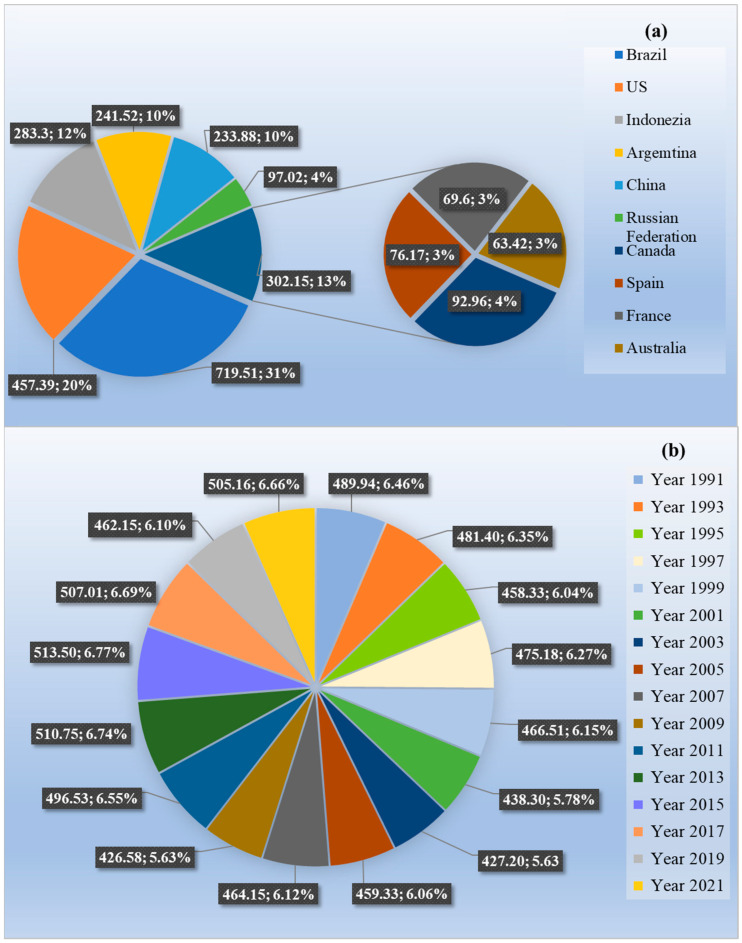
(**a**) The first 10 countries in the world in terms of the agricultural consumption of pesticides (2021). (**b**) Pesticide consumption in Europe (1991–2021) (thousand metric tons and percentages) [39,40].

According to the latest reported data, at an international level, in 2021 Brazil was the nation that used pesticides the most (719.51 thousand metric tons), followed by the United States (457.39 thousand tons) (Figure 3a) [39]. Regarding Europe, Spain and France occupied the eighth and ninth places, with 76.17 and 69.60 thousand metric tons [39]. Moreover, 505.16 thousand metric tons of pesticides were used in Europe, accounting for 14% of global usage (Figure 3b) [40]. It was observed that between 1990 and 2021, pesticide usage had globally increased by 96% [39].

Pesticide residues were detected in European agricultural soils due to their intensive usage, but also to their persistence [41]. As a result, many are bio-accumulative and toxic, and are considered potential priority pollutants [42].

Various studies revealed the presence of pesticides in soils. For example, from 180 samples of soils from France, 27 pesticides were detected [43,44,45]. It is obvious that higher concentrations of pesticides were detected in soil samples from conventional than from organic agriculture. Generally, mixtures of fungicides, herbicides, and insecticides were detected in soils. It is from these that the risk of pesticide mixture residues to living organisms arises, as well as the possible outcomes for the entire ecosystem [44].

The retention of pesticides in soils is predominantly governed by adsorption, with several feasible mechanisms: charge transfers, hydrogen bindings, interactions with metallic cations, polar interactions, or Van der Waals forces [46]. Generally, the pesticide adsorption is directly dependent on the volume and on the branching degree, related to the surface [46]. Pesticide persistence and mobility are governed by their characteristics. These are, in turn, influenced by soil environment, weather, or application method. The nature of the atoms or functional groups dictates the pesticide’s electronic nature, and consequently their permanent dipole moment and polarizability. These factors determine the interaction type between pesticides and soil (for example donor–acceptor electrons or hydrogen bonds. Ionization influences the pesticide’s charge, in the case of weak acids or bases depending on the soil pH and on pK_a_ (acid dissociation constant) or pK_b_ (base dissociation constant) values [46]. For example, one of the most intensively applied herbicides, glyphosate, has a pK_a_ = 4. In this respect, its sorption increases if the soil pH decreases, and the number of the herbicide’s negative charges decreases [46].

The soil components mainly involved in the pesticide’s adsorption are clays (as silicate minerals), hydroxides, or oxide, their surfaces being primarily hydrophilic due to the presence of hydroxyl groups or exchangeable cations [47]. Organic matter represents a percentage of the total dried material in soil; however, it represents an important pesticide adsorbent due to its high reactivity. The organic matter adsorption capacity is influenced by its chemical composition and, also, by its size, having an increased number of sorption sites related to a higher surface area [46]. The soil pH is important mainly for the adsorption of ionic pesticides such as glyphosate, its sorption increasing once the soil pH decreases [47]. Parameters that influence the soil structure include bulk density and pore geometry, which depend on agricultural practices as well as climate. Pesticide migration through aggregated soils is primarily determined by kinetic sorption and diffusion: in a static regime, the adsorption rate decreases with the increase in soil bulk density, and in a dynamic regime, pesticide retention is influenced by pore water velocity or residence time [46].

The highest concentrations of pesticide residues in soils were detected for glyphosate and its AMPA (aminomethylphosphonic acid) metabolite (herbicide), imidacloprid (insecticide), and boscalid (fungicide), but also various azole fungicides (such as epoxiconazole and tebuconazole) [41,48]. It was observed that the concentrations sometimes surpassed their predicted environmental concentrations (PEC), but they were lower than their toxic concentrations determined for various spikes on soil invertebrates [44]. Nevertheless, when these pesticides are mixed, their environmental risk to soil can be higher than the risk caused by single-chemical exposure [49].

Even though the actual regulations include banned pesticides, they only mention a single-chemical risk perspective. The recent discovery of pesticide mixtures’ negative effect on non-target fauna encourages the idea that they should be consistently monitored, their cumulative risks being taken into consideration.

To observe the effects of pesticides in soil, invertebrates were used as indicators in ecotoxicity tests. These soil invertebrates have been affected by exposure to pesticides [50]. Various soil invertebrates, belonging to the same taxonomic group, will not manifest the same sensibility to pesticide residues [50,51,52]. For example, it was demonstrated that *Aporrectodea caliginosa* and *Lumbricus terrestris* were more susceptible to pesticides than *Eisenia fetida*, although all of them belong to the earthworm class [50]. Also, *Eisenia andrei* and *Folsomia candida* manifested the highest sensitivity to neonicotinoids [51].

One of the most recent assessments for the determination of pesticide mixture toxicity is the use of biochemical biomarkers [53]. Acetylcholinesterase (AChE), carboxylesterase (CbEs), and glutathione-S-transferase (GST) represent specific target enzymes that can reveal the sensitivity of organisms when they are exposed to carbamates or organophosphates [50]. In this way, a combined collection of test species and biochemical procedures will highlight the ecotoxicological responses to pesticide mixtures within the environment.

This overview presents more than 50 studies and surveys concerning the fate of pesticides. It was observed that the pesticide sampling from soils or detection procedures were not homologated, probably because a unified sampling and monitoring protocol is not currently available [53]. However, it must be emphasized that the scope of this overview was not the analytical chemistry of pesticides in soils, the aspects regarding extraction and analytical instrumentation being only briefly mentioned.

The period between land treatment with pesticides and soil sampling represents a key issue, the time directly influencing the pesticides’ chemical degradation. Various studies indicated the sampling period, a quarter of them reporting that the sampling was achieved during harvesting (spring–summer) [41,54,55], and another 20% reporting sampling in autumn–winter [56,57].

Many studies only considered the upper layer of the soil (from 0 to 30 cm), which is considered the most relevant for the accumulation of pesticides, being related to ploughing [54,58,59]. Various depths were considered for sampling from this layer: up to 5 cm [55], up to 10 cm [60], up to 15 cm [61], and up to 20 cm [43]. Leaching represents pesticide transport downward in ground water and it is determined by principles of mass transfer and molecular diffusion. The groundwater is polluted due to pesticides leaching from soil. The more pesticides are removed from the soil, the less pesticides will leach [47,59].

However, there are several studies that approached deeper samplings: up to 60 cm depth [62,63], up to 100 cm depth [64], and even up to 300 cm [65]. The sampling consisted in obtaining several subsamples spatially distributed within the studied area, from three [48] to fifty [66]. The sampling instrumentation included a stainless-steel spade and soil auger, deeper samplings being achieved using soil samplers such as a soil auger [67], or soil drilling equipment [68]. The universal storage of the soils was in plastic bags. However, other storage containers were also used, for example glass containers [69,70], aluminium boxes [71], or stainless-steel containers [54]. After being delivered to the laboratories, the samples were dried in air and sieved before being processed. Many of the reviewed studies did not provide details about the sampling tools and methods used.

Regarding pesticide extraction, the most used procedure was extraction with an organic solvent (acetonitrile, hexane, or methanol), or a mixture of solvents. Another pesticide extraction method was the QuEChERS procedure (quick, easy, cheap, effective, rugged, and safe) [54].

Regarding pesticide analysis, chromatographic methods represent the more efficient ones for multi-residual pesticide determination [54]. Both gas and liquid chromatography were applied for both qualitative and quantitative determination of pesticides from soil [54]. Recently, liquid chromatography was used for comparing with earlier methods [54]. Various researchers used both methods in order to determine as many pesticides as possible [72,73]. More recently, mass spectrometry has been intensively applied by coupling it with gas and liquid chromatography, in order to improve pesticide detection [74].

Regarding the European monitoring of pesticides in soils, the most comprehensive investigation took into consideration glyphosate and its metabolite and another 74 pesticides within eleven European countries [41]. A total of 317 soil samples were investigated for pesticide residues in soils; in 83% of them at least one type of pesticide residue was present [41]. The most detected pesticides were boscalid (28%, up to around 400 μg/kg), glyphosate metabolite-aminomethylphosphonic acid (41%, up to about 1900 μg/kg), epoxiconazole (25%, up to 150 μg/kg), glyphosate (20%, up to 2050 μg/kg), and tebuconazole (13%, up to about 200 μg/kg) [41,75]. Regarding the European regions, it was observed that from the eastern soils, 93% contained pesticide residues, the highest one from all of the studied regions [41,75]. The southern region presented the lowest detection frequency of pesticides, but with high values for glyphosate and its metabolite (up to 2000 μg/kg and 1900 μg/kg, respectively). The lowest detection frequency for glyphosate was registered in Poland (7%), the highest one being determined in Portugal (53%). Regarding the glyphosate metabolite, the lower detection frequencies were registered in Italy and Greece (around 17%) and the upper one was registered in Denmark (80% of the soil samples) [41,75]. After observing these results, it can be concluded that 100% of the soils from root crops contained pesticides, glyphosate and its metabolite being higher in regions with permanent and root crops (30% and 50%, respectively) and lower in dry and fodder crops (5 and 30%, respectively) [54].

A total of 26 of the used pesticides and their transformation products were investigated in 105 soils in northern France, with nine compounds being detected [76]. A total of 29% of soils contained methabenzthiazuron (up to 0.05 μg/kg) and 21% of them contained prometryne (up to 8.5 μg/kg). The highest detection frequency was reported for atrazine (80%, up to 0.15 μg/kg), diuron (55%, up to 3.5 μg/kg), isopropylphenyl methylurea (70%, up to 0.18 μg/kg), isoproturon (65%, up to 1.18 μg/kg), and linuron (60%, up to 6.3 μg/kg) [76].

It is worth mentioning an older investigation from Spain on the occurrence of herbicides in the soils of three citrus orchards having a long-documented herbicide application history [77]. The herbicides residues (from 10 to 600 μg/kg) showed that, except for a few cases (where high concentrations of atrazine, bromacil, and diuron were detected), the herbicides’ accumulation was low even after a long duration of use. Soil samples from Spanish cropland were investigated for herbicides at different intervals after application, the main pesticides being atrazine (up to 40 μg/kg), ethalfluralin (up to 50 ethalfluralin), and terbuthylazine (550 μg/kg) [78]. Twenty-six soils were sampled from the southern region of Spain, with 126 pesticides being detected [79]. A total of 50% of the soil samples contained pesticides, the most frequent one being dimethomorph (up to about 90 μg/kg). Other detected pesticides were bifenthrin, bupirimate, fludioxonil, and tetraconazole [79]. A total of 31 soil samples were taken from eastern Spain to investigate pesticide occurrence, with the highest detection frequency and concentration being for chlorpyrifos (80%, up to about 60 μg/kg) [54,80].

A total of 18 conventional agricultural sites were studied in northern Portugal, with eight pyrethroid pesticides being monitored in summer and winter [81]. The only one detected was deltamethrin, in 8% of soil samples up to 102 μg/kg, but only in a summer sampling campaign [81].

Some researchers periodically monitored 34 soils in the Czech Republic to assess temporal and spatial pesticide occurrence, with 24 being detected in all of the sampling intervals [82]. A total of 19 targeted chemicals were not detected in the soil samples probably because they were banned in the meantime (such as atrazine or acetochlor), or because they had short half-lives (for example, 2,4-dichlorophenoxyacetic acid) [82]. The pesticides having the highest concentrations were diflufenican (up to 150 μg/kg), chlorotoluron (up to 90 μg/kg), pendimethalin (up to 300 μg/kg), and tebuconazole (up to 150 μg/kg) [82]. An older investigation was performed for 75 agricultural soils from the Czech Republic, for the detection of 68 pesticides [56]. A total of 99% of the soils contained residues from at least one pesticide, with 81% of samples containing at least one pesticide above the national generic soil limit of non-chlorinated pesticides (0.01 mg/kg) [54]. Additionally, the occurrence of chlorotriazine herbicides was investigated, terbuthylazine being present in 17% of the samples and with a concentration of up to 38 μg/kg, while simazine, after ten years of being banned, was found in only one sample (9 μg/kg), atrazine being absent in all analysed samples [57]. A total of 25% of the samples contained up to 75 μg/kg chloroacetanilides (for example, S-metolachlor o metazachlor) [57].

Hungary maintains one of the most important soil monitoring programs within the European Union, with over one thousand two hundred soils sampled after 1999, monitored for forty-seven pesticides [83]. It was observed that 18% of the soils sampled between 2008 and 2013 contained detectable levels of pesticides [64]. The detected pesticides were atrazine (up to around 600 μg/kg), acetochlor (up to 80 μg/kg), and trifluralin (up to 200 μg/kg) [68].

A total of 20 soils were sampled in Romania from lands cultivated with fruits and vegetables in order to detect the presence or absence of 70 pesticides [84]. None of the currently available pesticides were detected, probably due to the deep sampling (up to 50 cm), which was conducive to low concentrations, as pesticides tend to accumulate in higher soil layers [52]. The consequence of this can be concentrations under the quantification limit of the method, in this case a limit of 10 μg/kg [84].

Studies regarding pesticide detection were also performed in Serbia, where 24 soils were sampled from farms cultivating fruits and vegetables, with 21 pesticides being monitored [85]. The detected pesticides included acetochlor (up to 13 μg/kg), atrazine (up to 0.05 μg/kg), chloridazon (up to 54 μg/kg), chlorpyrifos (up to 47 μg/kg), fenitrothion (up to 81 μg/kg), fluorochloridone (up to 45 μg/kg), napropamide (up to 28 μg/kg), and prometryn (up to 54 μg/kg). The concern came from the fact that, although they were detected, each farm declared that no chemicals were used [85]. The conclusion was that the residues came from previous pesticide application, due to their high persistency in the soil [85].

Although the above-mentioned results provide information about pesticide persistence, further in situ and ex situ soil monitoring is a must to assess an authentic frame of reference. Moreover, polluted soils with pesticides should be subjected to remediation before further use. The selection of the remediation method will depend on the removal efficiency and on its sustainability. It was demonstrated that the electrokinetic (EK) remediation method represents an environmentally friendly and economic choice, the main advantage being the simultaneous removal of both inorganic and organic contaminants [86]. The data limitation for real contaminated soils influences the implementation of this technique on a large-scale. Another remediation method is ex situ soil washing, which can remove pesticides using liquids containing aqueous solutions of different extractants [87]. Soil flushing represents an in-situ remediation method involving an aqueous solution (acids, bases, cosolvents, oxidants, surfactants, solvents, or water) injected into contaminated soil to increase solubility. The contaminated fluids are then captured and pumped to the surface using standard wells, the desorbed contaminants being further treated. The method’s efficiency depends on hydrogeological parameters, such as soil moisture or type, but also on the pesticide type [87]. This conventional technique may be applied to remediate soil contaminated with pesticides (such as atrazine or phosalone), but it is not currently used. Biosurfactants were also considered for pesticide biodegradation in soil [87]. Their usage increases hydrophobic pesticide solubility by elevating the surface area, and increases the emulsification, resulting in the dissociation of pesticide molecules, which are bioavailable for microbes to process them [87]. Advanced oxidation processes (AOPs) represent techniques in which soil is mineralized, pesticide residues being then transformed into water and carbon dioxide using oxidant reagents (chlorine, hydrogen peroxide, hypochlorite, or ozone) [88].

Bioremediation and bio-stimulation have recently been considered due to their reduced costs in removing pesticides, making them economically and technically viable for large-scale application. It is a slow process that requires specialized bacteria to accomplish optimal decomposition [89]. Bio-stimulation implies adding nutrients and other complementary compounds to stimulate local microbes’ rapid proliferation [87]. These methods are influenced by several parameters, such as environmental constraints, microbial ecology, or pollutant types [87]. Some of the problems that typically appear when these methods are applied alone can be overcome by combining phytoremediation with bio-augmentation [90]. Generally, biological remediation techniques are considered environmentally friendly because they transform pesticides into less harmful compounds. Nevertheless, they are strongly influenced by various factors, such as organic loading, oxygen levels, pH, or temperature. Sustainability in pesticides removal and soil remediation must consider optimizing energy and resource usage, guaranteeing the long-term effectiveness of pesticide reduction. Life cycle assessment (LCA) and economic feasibility are critical, and outreach and awareness campaigns can inform farmers, customers, and the public about the environmental effects of pesticide overuse. These concerns are critical for addressing pesticide contamination and for preserving a healthy environment for future generations.

## 4. Pesticide Residues in Fruits and Vegetables

**Figure 4:**
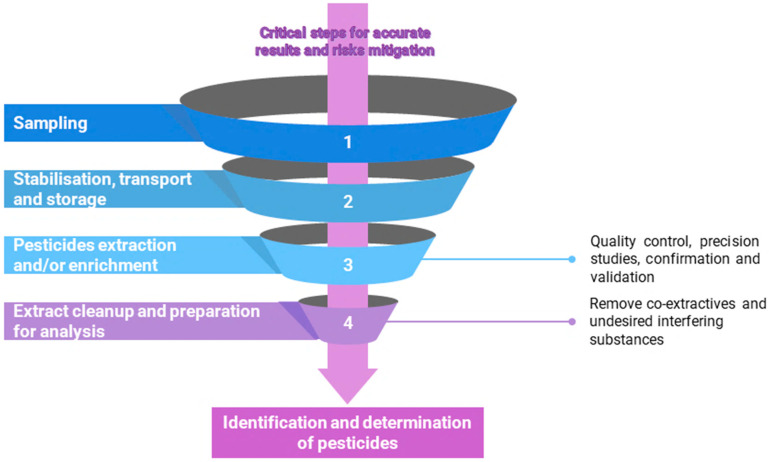
The main phases for determining pesticides in fruits and vegetables.

Despite the progress in developing preparation procedures to efficiently analyse pesticides, their determination in biological samples remains a challenge. Various problems arise within pesticide residues analysis: matrix complexity and diversity within biological materials, or the low pesticide concentrations in fruits and vegetables samples [91]. Therefore, targeted compounds must be isolated from the matrix and then they must be enriched before analysis [91]. The entire procedure for pesticide residue determination is complex and includes various phases summarized in Figure 4.

It is worth mentioning that these phases as well as the entire procedure must be validated to guarantee the requirements and demonstrate its fitness.

The preparation of the sample clearly affects the result, simultaneously being important the homogeneity and representativeness of the sample [91]. An important factor influencing the results is also the sample storage, including the samples being frozen and kept in darkness. According to some authors, in the case of fruit and vegetables, prior to other sample preparation steps, surface contaminants must be removed by washing them with deionized water, but this practice is wrong, as it may remove surface residues. Then, the samples can be dried at higher or ambient temperature, crushed, or ground, after which the samples must be homogenized [91]. The procedure for the sample preparation must be adapted according to the sample type so that it does not cause any analyte loss or additional contamination [91].

An essential phase is the next one, consisting in the isolation and/or enrichment of the targeted analytes. This step is necessary due to potentially low pesticide concentrations. This stage consists of shifting the analytes from the first matrix to a second one, simultaneously removing the interferences and increasing the targeted analyte concentration above the quantification limit of the analytic method used. This procedure is often used due to low pesticide concentration in fruit and vegetables, the solid matrix being replaced with a liquid one [92,93]. To achieve this, an appropriate extraction must be applied: liquid–liquid extraction, ultrasound-assisted extraction, Soxhlet extraction, microwave-assisted extraction, supercritical fluid extraction, or membrane extraction. Nevertheless, high amounts of toxic solvents will be used [91].

One of the new extraction methods includes the Soxtec method, which provides better recoveries and is faster than the traditional Soxhlet one, multiple samples being simultaneously extracted. However, the instrument is an expensive one. Solvent-extraction methods are often assisted by ultrasounds or microwaves for increasing the analyte recovery. The most used solvents include acetone, acetonitrile, or ethyl acetate. Table 3 presents some of the solvents used for various pesticides’ extraction from fruits and vegetables.

The analyte isolation from fruits and vegetables also includes a clean-up step, which assures an increase in the targeted analyte concentration and minimizes the interference, such as that from chlorophyl, fats, or sugars [91]. The most commonly used techniques include adsorption chromatography, gel-permeation chromatography, matrix solid-phase dispersion extraction, solid-phase extraction, solid-phase microextraction, and stir-bar sorption extraction [95].

Solid-phase extraction (SPE) represents the most used one from the above-mentioned methods [104]. Briefly, the sample flows through a column filled with an appropriate sorbent, the targeted analytes being adsorbed by the sorbent; finally, the retained analytes are recovered using a specific solvent. Typical sorbents are C18 (octadecyl alkyl substituen), graphitized non-porous carbon, polymers, or ion exchangers. This technique proved to be simple, automated instruments having been developed [94,103]. In solid-phase microextraction, the analytes are adsorbed on a fibre covered with an appropriate solid phase that is flushed from a micro-syringe [105]. The analytes are then thermally desorbed and sent to the analysis instrument. The advantage of this method consists in the possibility of eliminating solvent usage [105].

Another reported clean-up method is gel permeation chromatography, a type of size-exclusion chromatography, which provides the separation of micromolecular pesticides from macromolecular compounds within the matrix [106]. However, it has a poorer resolution compared to adsorption methods, mainly when gradient-elution is applied. Quick, easy, cheap, effective, rugged, and safe (QuEChERS) methods have been developed for pesticide determination techniques [107]. They represent a combination of liquid–liquid and solid-phase extraction. The advantage consists of minimum consumption of sample and toxic solvents. When the QuEChERS method is applied for pesticides determination in fruit and vegetables, matrix effects are mostly removed and an increased targeted analyte concentration can be accomplished.

The final phase in pesticide determination represents their actual identification and quantification using appropriate equipment. The usual one includes gas chromatograph and high-performance liquid chromatography. Gas chromatography can be applied for the determination of pesticides that are volatile and thermal stable [92]. The selection of chromatographic column is of ultimate importance for a good analyte separation, as well as for qualitative and quantitative analysis. The multi-residue pesticides in fruits and vegetables can be determined using gas chromatography coupled with mass spectrometry, due to the efficient chromatographic separation, good sensitivity, and confirmation based on mass spectra. Nevertheless, liquid chromatography coupled with mass spectrometry provides a rapid and effective determination of various pesticides or metabolites rarely investigated in food or difficult to determine by gas chromatography [108]. In order to determine various types of pesticides from fruits and vegetables, several detection systems were applied: an electron-capture detector highly sensitive to pesticides containing electronegative atoms, mainly halogenated ones; mass spectrometry for various pesticides determination; a flame-photometric detector used for other organophosphorus pesticides; a nitrogen-phosphorus detector used to simultaneously determine organonitrogen and organophosphorus pesticides; and a thermionic specific detector used to determine both organonitrogen and organophosphorus pesticides.

In order to determine organonitrogen and organophosphorus pesticides from fruits and vegetables, other techniques, such as the chemiluminescence method [109] and gas liquid chromatography can also be applied [110].

The members of the European Union must organize efficient campaigns to monitor food quality, to ensure its safety. Regarding pesticide residues, this assignment is achieved by organizing food monitoring and official inspections.

Table 4 lists data regarding the determination of pesticides from fruits and vegetables.

Analysing Table 4, it can be noted that several studies are older than 10 years. Techniques such as GC-FCD were replaced with GC-MS or LC-MS/MS methods to increase the performances [114].

Analysing the literature, it was obvious that there is no one universal procedure for an effective determination of pesticide residues. The researchers continuously look for a method that can simultaneously provide the best analyte recovery and the lowest possible limit of detection/quantification. Gas chromatography coupled with mass spectrometry was used for the determination of 17 pesticides from 240 samples [98]. No pesticide was detected in 67% of the samples, 25% contained pesticides under the regulation limits, and 8% of samples contained pesticides above the limits. More important was that 5% of the samples contained five or more pesticides [98]. For example, grapes and sweet peppers contained 10 pesticides and strawberries contained 14 [98].

Tandem mass spectrometry ensures improved selectivity and sensitivity. Ions separated by the first detection system are then fragmented, the derivative ions being analysed by the second one. The effect is that the background is reduced, the signals being enhanced and the detection limit being lowered [91].

Two-dimensional gas chromatography improves peak resolution and decreases the matrix interferences [115]. Each system must have two columns with various retention mechanisms, inter-connected by a modulator for collecting or sampling fractions from the first column then injecting them into the second one [116]. The advantage consists in the fact that the two columns have an independent separation mechanism, but it can also simplify the fruits and vegetables sample preparation.

Fast gas chromatography was applied to decrease the analysis time and improved peak resolution. When compared with the classical method, fast chromatography needs shorter capillary columns with smaller diameters, solid phase films with an average thickness of about 0.1 µm, higher pressure, and faster flow rate for the gaseous carrier [91,117]. The result is an improved precision of the method [91,116].

Reversed-phase chromatography has been recently applied for the separation of less polar pesticides, relying on target pesticide derivatization and stationary phase modification [118]. The derivatization reagents interact with the analyte and a nonpolar modified group, enhancing the retention of the derivative highly polar pesticides. For example, the fluorenylmethyl chloroformate (FMOC-Cl) derivatization reagent was used after a solid-phase extraction method to determine glyphosate and aminomethylphosphonic acid residues from 16 food matrices. In this way it was demonstrated that the derivatization is a valuable method to determine low concentrations of pesticides in foods [118].

LC-MS represents the most intensively applied technique for the analysis of pesticide residues. LC tandem MS (LC-MS/MS) has been used to quantify highly polar pesticide residues in food matrices, the standard method being electrospray ionization combined with a triple quadrupole mass spectrometer (QqQ MS) [118]. According to the QuPPe-PO method (Version 12), 55 highly polar pesticides are currently tested, 33 of them being analysed in the positive mode and 22 in the negative mode [119]. In the positive ion mode, azo compounds with the N atoms acidified (using acetic acid or formic acid) can be determined, forming [M+H]^+^; in the negative ion mode, phosphoric acid derivatives can be analysed, forming [M−H]^−^ [118]. To date, although other ionization methods such as APCI have been applied, ESI remains the most used ionization method in pesticide analysis.

Multiple reaction (MRM) and single reaction monitoring (SRM) represent the most powerful scan modes for pesticide quantification. For example, MRM was applied to determine eight highly polar pesticides in cherries, resulting in good repeatability and reproducibility [120]. Recently, parallel reaction monitoring (PRM) in quadrupole-orbitrap MS has been considered an alternative for pesticide quantification. It overpasses the disadvantages of MRM or SRM scan functions, such as low resolution and an impaired sensitivity [118]. The PRM scan function was coupled with a solid-phase extraction method and a dilution sample pre-treatment method to quantify pesticide residues in food; nevertheless, the narrower dynamic range constraints are less applied than in the MRM [121]. High-resolution mass spectrometry (HRMS), including orbitrap and time-of-flight (TOF), has been used for pesticide qualitative determination. Due to HRMS’s high mass accuracy, the full scan mode can determine pesticide residues by their accurate mass [118]. All ion fragmentation (AIF) was used to determine seven pesticides in fruits and vegetables, obtaining reliable results considering that at least two fragments were monitored per pesticide [122].

The actual trend is the development of analytical techniques that can detect and determine multiple analytes in a single run, the encountered problem being that the monitored compounds can be at low concentrations, having various physicochemical characteristics.

## 5. Solutions to Mitigate Pesticides Residues from Fruits and Vegetables

As it was already mentioned, pesticides are intensively used during fruit and vegetable production to maintain the expected quantity. As a consequence, their residues remain in the raw agriculture commodities, having a negative impact on living creatures and the environment [123]. These residues have various action mechanisms (systemic or contact), acting as cancerogenic, mutagenic, neurotoxic or teratogenic compounds [108]. The International Agency for Research on Cancer (IARC) categorised agents as potentially carcinogenic to humans (Group 2A) if the carcinogenic potential was demonstrated in animal model systems, but human statistics are not yet convincing. Group 2A contains six pesticides (captafol, diazinon, 4,4′-dichlorodiphenyltrichloroethane-DDT, dieldrin, glyphosate, and malathion) [124]. Although these pesticides were mainly banned within the EU and various other countries, many countries in the world still use them as pesticides or for other special purposes [124].

Concerning food safety, fruits and vegetables containing two or more pesticides or their metabolites, having or not having the same mechanism on humans, must be considered to be of potential toxicological interest. It must be stated that consumers, in addition to raw fruits and vegetables, also eat their processed products, concentrates, jams, juices, purees, or pastes. Food processing can induce an increase or decrease in the pesticide residues in the final product, this being one of the modalities to eliminate them and to mitigate their risk to human health [109]. Various studies reported that pre- or post-food processing may reduce the load of pesticides in the final product [125,126,127].

**Figure 5:**
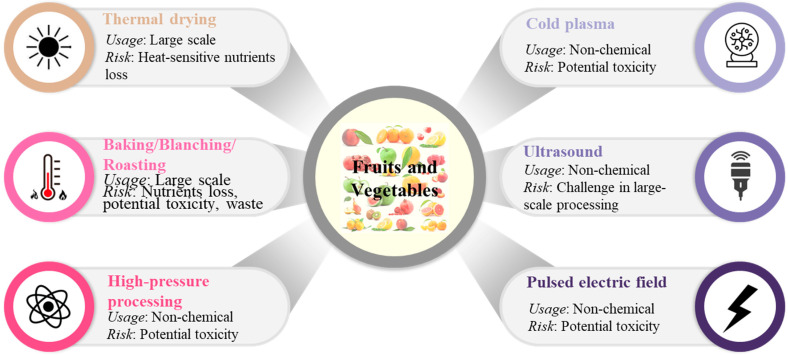
Methods for reducing pesticide residues in fruits and vegetables [112].

Food processing can be used to increased safety or enhance food quality [128]. Food processing can be classified into non-thermal (such as cold plasma, fermentation, high-pressure processing, ozone, pulsed electric field, radiation, or ultrasound) and thermal (such as baking, blanching, boiling, cooking, drying, roasting, and sterilization) processing methods (Figure 5) [128]. It must be stated that most of these methods are only in experimental stages. However, several factors can affect the persistence of pesticides in fruits and vegetables, for example, the pesticide physicochemical characteristics (such as the octanol–water partition coefficient, water solubility, vapour pressure, or volatilization), the fruit or vegetable variety, as well as the climate [128].

It must be stated that imposed regulations are the first step in controlling the use of pesticides to diminish their adverse effects [129]. Washing, peeling, or milling can partially remove pesticide residues, but the consumers or industries do not entirely put into application these simple steps while transforming the food into the desired product [130]. Furthermore, the application of these steps is dependent on the final product type.

There are few techniques that have been applied to remove pesticides or their metabolites from food products [131]. For example, chlorine water or detergents can be used to reduce the pesticide residue content. Another method consists in thermal treatments, such as blanching or sterilization, this technique being considered safe for health. However, its disadvantage is that it influences the colour, flavour, or texture of the food products. In this respect, efficient methods with minimum effect on the environment and which do not influence the food product characteristics must be developed [132,133], this review providing an overview of the current techniques applied for pesticide residue degradation.

Peeling represents one of the most important operations used for pesticide mitigation in fruits and vegetables. Pesticide residues usually accumulate on the product surface and can be totally removed by peeling [134]. This operation can be applied for fruits or vegetables like banana, mango, orange, pineapple, or watermelon [135]. The peeling process effectively removed 67% of diazinon residue from cucumbers. By this process, 100% of acrinathrin and azoxystrobin, and 90% of kresoxim-methyl residues were removed from zucchini [136]. Pesticides can transfer and accumulate in fruits and vegetables through two main pathways: external and internal exposure [135]. In external exposure, pesticides enter the epicarp or leaf and then transfer to the pulp. Internal exposure takes place when pesticides from soil or water are absorbed by the plant roots and distributed all over via circulation. Different parameters, such as cultivation conditions, plant species, and soil type, as well as the physicochemical properties of pesticides, impact the uptake of pesticides by plants [137].

Boiling, cooking, or sterilization treatments can help the reduction of pesticide levels within various food products. It was reported that sterilization at 120 °C for 15 min reduced 65% of maneb residues from tomatoes [138]. However, the thermal degradation of maneb may induce the production of a toxic metabolite, ethylenethiourea. Also, it was demonstrated that the boiling procedure applied after washing induced the reduction of fenazaquin residue from okra fruits by 40% [139].

Various chemical compounds have been applied for the pesticide’s removal from food. For example, chlorine dioxide or photocatalysts were used to reduce cyprodinil, iprodione, and tebuconazole from apricots, nectarines, or peaches (Supplementary Materials, Table S1) [132]. The chlorine dioxide (chemical compound with the formula ClO_2_) removed 60% of the tebuconazole, while photocatalysts removed 50–70% of the pesticide residues from fruits [132]. Other studies demonstrated that rinsing with detergents, followed by peeling or household preparation can reduce the captan residue from apples and profenofos residue from vegetables (Supplementary Materials, Table S1) [140,141]. The used washing solutions included acetic acid and soap, and household preparation included blanching and frying.

A washing procedure (using sodium hypochlorite and peroxyacetic acid) combined with ultrasonication was applied to reduce pesticide residues from various fruits and vegetables. It was demonstrated that sonication improved the efficiency of the removal (Supplementary Materials, Table S1) [142]. Also, washing solutions and ultrasounds reduced the organophosphorus pesticide residues from cucumbers (Supplementary Materials, Table S1) [143].

Ozonification represents an efficient, economic, and feasible procedure that can be used for pesticide reduction [144]. Currently, ozone is applied for fruit and vegetable storage, possessing high air stability (Supplementary Materials, Table S1) [145]. Ozone treatment (3 mg/L) proved to be efficient in the removal of around 80% of the pesticide residues from tomatoes [146]. Ozone was also used to remove pesticides like difenoconazole and linuron from carrots, the treatment being efficient, without the formation of toxic intermediaries (Supplementary Materials, Table S1) [147]. The influence of ozonification on the removal of cypermethrin and chlorpyrifos from spiked tomatoes was studied; after 30 min, the maximum reduction of 98% chlorpyrifos and 87% cypermethrin was observed, demonstrating the ozonification efficiency [148]. The ozone positively influenced the removal of chlorfenapyr residue from tomatoes [148]. The maximum removal yield was obtained after only 15 min [148].

Irradiation represents a popular method applied for food preservation [149]. The irradiation method was applied for cypermethrin, malathion, and pirimiphos-methyl reduction from fruits and vegetables [150]. The 1 kGy dosage irradiation of potatoes yielded 18% removal for pirimiphos-methyl residue, the pesticide removal from grapes being at moderate levels at 7 kGy irradiation dosage [150]. The effect of gamma irradiation was investigated on the reduction of azoxystrobin and carbendazim from strawberries, the efficiency increasing with the adsorption dose increasing, due to the formation of reactive radicals which favoured the radical–radical recombination (Supplementary Materials, Table S1) [151]. Gamma irradiation was used for carbamate and organophosphorus pesticide removal from vegetables, their concentrations decreasing with increasing irradiation dosage (Supplementary Materials, Table S1) [152]. The highest yield (90%) was obtained for diazinon at 1 kGy, and for chlorpyrifos, with 80% being removed. Electron beam irradiation was used for peas profenofos reduction, the highest yield (48%) being obtained at 32 kGy [131].

The pulsed electric field technology represents a new approach with encouraging results for food [153]. Its degradation mechanism involves polar molecule rotation and vibration, followed by oxidation via hydroxyl radicals [148]. It was tested on the removal of pesticides (such as chlorpyrifos and methamidophos) from apple juice, revealing that the process parameters (applied electric field strength and pulse number) had a direct influence on the removal of pesticide residues [154]. The best results were obtained when 6–26 pulses and 8–20 kV/cm electric field strength were applied, probably due to the enhancement of polar molecule rotations and vibrations, accelerating in this way the pesticide disintegration. Pulsed electric field application also reduced cyprodinil, procymidone, pyrimethanil, and vinclozolin pesticides from dry white wine [155]. These pesticide residues were significantly reduced after a pulsed electric field application with 5–20 kV/cm for up to 2 ms, the residues mitigation reaching 50% for cyprodinil, 25% for procymidone, about 25% for pyrimethanil, and around 30% for vinclozolin [155].

Cold plasma is an emerging technology used for industrial food, involving food exposure to ionization radiation [148]. The oxidation reaction of pesticides with free radicals and reactive species leads to their decomposition into intermediary or less toxic chemicals. Cold atmospheric plasma was applied to remove pesticides like boscalid and imidacloprid from blueberries (Supplementary Materials, Table S1) [156]. The removal efficiency reached 80% for boscalid and 75% for imidacloprid, with a treatment 80 kV for 5 min that led to an increased number of reactive species [156]. The influence of dielectric barrier discharge plasma on chlorpyrifos removal from tomatoes was studied [142]. The maximum removal of 89% was obtained after only 5 min for peaches (Supplementary Materials, Table S1) [157]. Cold plasma was used to reduce pesticides from mangos (Supplementary Materials, Table S2) [158]. The plasma procedure induced a reduction of around 65% cypermethrin and 75% chlorpyrifos from mangos through atomic oxygen and hydroxyl r with the gaseous argon flow rate of 5 L/min for 5 min being the best for an efficient removal of pesticide residues [158]. Gas-phase surface discharge plasma helped with organophosphorus pesticide elimination from *Lycium barbarum* berries, the removal efficacy depending on the exposure interval and the applied voltage for peaches (Supplementary Materials, Table S1) [159]. The plasma generated reactive oxygen species that contributed to dichlorvos and omethoate degradation into smaller and less toxic compounds, 97% dichlorvos and 99% omethoate being reduced after 30 min at 10 kV [159]. The atmospheric air plasma can efficiently remove pesticide residues from fresh apples (Supplementary Materials, Table S1) [160]. The approached procedure reduced 96% of the paraoxon from apples [160].

Recently, combined technologies have been efficiently applied for pesticide elimination, but only at laboratory scale [128]. Moreover, important disadvantages must be noted, such as elimination mechanism and degradation pathway, which have still not been elucidated, but also the increased cost. Thus, further research is needed for the combined technologies application.

## 6. Toxicological Insights and Risk Assessment of Pesticides

Toxicology can be defined as that science that studies the harmful effects of chemical substances on organisms, as well as their interactions with living organisms. It also deals with the identification, isolation, and determination of toxic substances, with their action on the body, as well as with the means used to combat toxic effects [161].

Particularly, ecotoxicology is the science that studies the interactions, transformations, and effects of chemical substances in the biosphere. The first steps in ecotoxicology were made towards the end of the second millennium when the negative impact of pesticides on the environment was highlighted [162]. Following this critical alarm signal, concern has increased both for deciphering the mechanisms by which the toxic effects are produced and for establishing the relationships that exist between the physicochemical properties and toxicity. Currently, special attention is paid to all ecosystem types, where the effects of pesticides on fauna, flora, and characteristic populations are studied [163,164,165,166,167].

### 6.1. Risk Assessment–Database Mining

In the EU, since 2006, through the Regulation (EC) No 1907/2006 of the European Parliament and of the Council of 18 December 2006 concerning the Registration, Evaluation, Authorisation and Restriction of Chemicals (REACH) [168], it has been established that the ECHA (European Chemicals Agency) manages the implementation of the REACH Regulation. The EU member states, as well as the stakeholders (e.g., products and articles manufacturers, producers and suppliers of substances and mixtures, etc.) should contribute sustainably to the promotion of alternative testing methods at the international and national level through the development and implementation of computational methodologies, in vitro methodologies, toxicogenomic tests, etc.

For database mining, the same pesticides investigated in Table 2 were considered as a case study.

**Figure 6:**
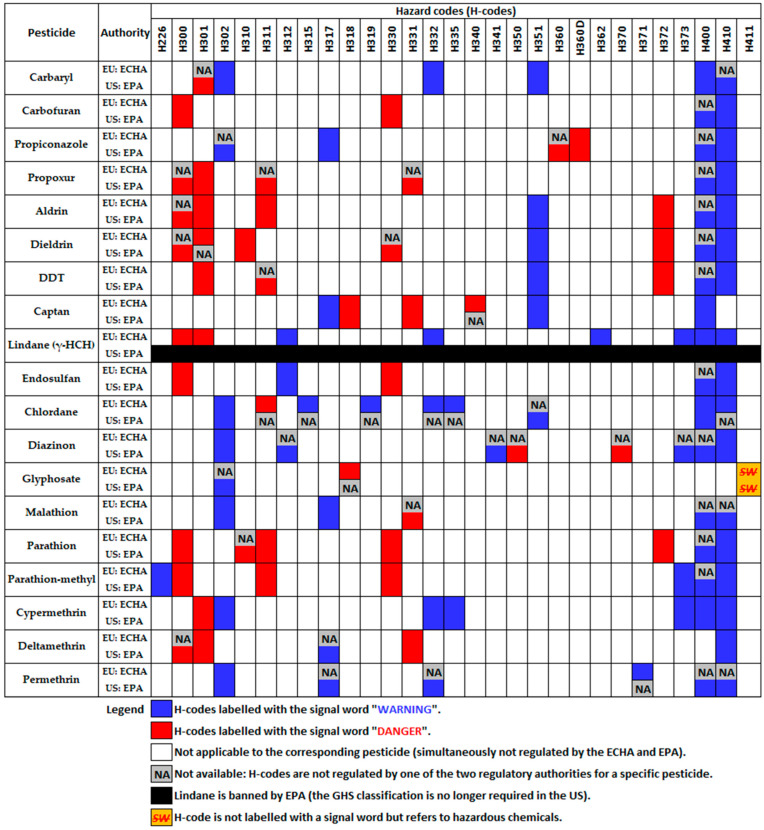
Current implementation of the GHS in the EU and US: co-occurrence of implemented H-codes and regulatory gaps between the ECHA and EPA.

Database mining was performed using the ECHA and EPA (U.S. Environmental Protection Agency) databases to evaluate the current implementation of the Globally Harmonized System of Classification and Labelling of Chemicals (GHS) of the United Nations (UN) [169,170] in EU and US. The UN GHS uses pictograms, hazard statements, and signal words to communicate hazard information on product labels and their corresponding safety data sheets. Results are briefly depicted in Figure 6, while the complete report is detailed in the Supplementary Materials, Table S2. Currently, for pesticides, the EPA still a simplified risk labelling system with a limited number of pictograms [171], while a White Paper was published for the adoption and implementation planning of the UN GHS (last updated on 26 July 2023) [172]. On the other hand, the ECHA uses a modified version of the UN GHS, approved by Classification, Labelling and Packaging (CLP) Regulation (EC) No. 1272/2008 (current version: 1 December 2023) [173].

From Figure 6 the huge regulations gaps between the EU and the US can be easily observed. Generally, the EPA assigned more H-codes to the investigated pesticides compared with the ECHA, with the notable exception of chlordane. Only cypermethrin has been assigned the same H-codes by the ECHA and EPA. A special case is that of lindane, as all of its forms have been discontinued (no longer produced or sold) in the US and it is banned for all types of uses and applications. Notably, only one pesticide, parathion-methyl is classified as flammable (H226). Moreover, the following series of remarks can be highlighted regarding pesticides labelled with the signal word “Danger” for various risks:

- Nine pesticides are currently classified as fatal if swallowed (H300–risk category 1 and 2): carbofuran, propiconazole, propoxur, aldrin, dieldrin, DDT, captan, lindane, endosulfan, chlordane, diazinon, glyphosate, malathion, parathion, parathion-methyl, cypermethrin, and deltamethrin;
- Eight pesticides are currently classified as toxic if swallowed (H301–risk category 3): carbaryl, carbofuran, propiconazole, propoxur, aldrin, dieldrin, DDT, captan, lindane, endosulfan, chlordane, diazinon, glyphosate, malathion, parathion, parathion-methyl, cypermethrin, and deltamethrin;
- Two pesticides are currently classified as fatal in contact with skin (H310–risk category 1 and 2): dieldrin (by ECHA and EPA) and parathion (only by EPA);
- Six pesticides are currently classified as toxic in contact with skin (H311–risk category 3): propoxur, aldrin, DDT, chlordane, parathion, and parathion-methyl;
- Two pesticides are flagged to cause serious eye damage: (H318–risk category 1: captan (by ECHA and EPA) and glyphosate (only by ECHA)) and (H318–risk category 2: one only by EPA (dieldrin) and another four by both authorities (carbofuran));
- Five pesticides are currently classified as fatal if inhaled (H330–risk category 1 and endosulfan, parathion, and parathion-methyl);
- Four pesticides are currently classified as toxic if inhaled (H331–risk category 2): two only by EPA (propoxur, and malathion) and another two by both authorities (captan, and deltamethrin);
- Captan is flagged by ECHA as it may cause genetic defects (H340–risk category 1);
- Diazinon is flagged by EPA as it may cause cancer (H350–risk category 1) and it causes damage to organs (H370–risk category 1);
- Propiconazole is flagged for reproductive toxicity as it may damage fertility or the unborn child (H360–risk category 1, by EPA), and it may damage the unborn child (H360D–risk category 1, by both authorities);
- Four compounds are jointly flagged by both authorities for causing damage to organs through prolonged or repeated exposure (H372–risk category 1): aldrin, dieldrin, DDT, and parathion.

Moreover, all of the investigated pesticides are flagged as environmental threats by at least one of the two authorities for similar types of hazards: H400 (very toxic to aquatic life, risk category 1), H410 (very toxic to aquatic life with long lasting effects, risk category 1), and H411 (toxic to aquatic life with long lasting effects, risk category 2).

Programmes to appraise the risks from dietary exposure to multiple pesticides have been around since the 1980s, considering the dose-addition of compounds from a mixture with similar bioactive characteristics that vary in their potencies. The main issue is to define a cumulative assessment class, which is contingent on a sound and high-quality toxicological database [174]. Due to the process complexity, harmonization has not yet been reached among regulatory organizations. Research from around the world has highlighted potential health risks from cumulative exposure only when very conservative premises were used [174].

Most investigations were performed using the probabilistic procedure for estimating the cumulative exposure, mainly using Monte Carlo Risk Assessment (MCRA). Various researchers appraised the exposure using the optimistic and the pessimistic procedure for the left-censored data [174,175]. Although the authors used the pessimistic technique only for registered pesticides, it was obvious that they over-evaluated the exposure and the risks. The concentration data from one of the investigations considered 135 of the 144 pesticides included in the cumulative assessment class (CAG), 126 being identified in at least one sample; over 99% of analysed matrices had no detected residues [175]. The EFSA (European Food Safety Authority) conducted a cumulative acute process for craniofacial alterations for the women-of-child-bearing-age citizens (abnormal skeletal evolution and head soft tissue changes/brain neural tube defects) [176]. The margin-of-exposure was higher than 100, but lower than 500, indicating potential health risks for a certain percentage of the people. These findings were highlighted when some conservative presumptions were considered, including imputing a concentration in water of 0.1 µg/L for the most powerful approved pesticides (with a concentration of 0.05 µg/L) and unit-to-unit volatility of five or seven (against a set value of 3.6) [174]. Most of the investigations employed national consumption data to estimate the cumulative dietary risks [174]. The used database included the GEMS/Food Consumption Cluster Diets for chronic exposure, reflecting food availability, not consumption. Despite the fact the FAO/ WHO Joint Meeting on Pesticide Residues applies these data to evaluate worldwide chronic dietary risks, they are not correlated to each country [174]. Moreover, the cluster diets target the general population and not a specific age class. The main provocation results from the availability of sound toxicological data to define the cumulative assessment class, and their scientific perception, which may vary among the researchers. To date, only retrospective cumulative evaluation has been assessed by regulatory organizations and the implementation of this procedure during pesticide registration and MRL establishment must be discussed with risk assessors and managers [174].

### 6.2. Risk Perception–Competitive Arbiter of Trust and Inclination to Buy Pesticide-Treated Greengrocery

Pesticides are considered as having positive effects on agriculture. However, an important question is arising: *How do end-users perceive this from a health and environmental point-of view?*

It is well known that people conscious of health risks will consider taking protective actions [177]. Furthermore, food safety risks lead to decreasing consumer willingness to purchase greengrocery [178]. To associate the objective risk conception (the probability of a threat appearance and its proportion) with the subjective conception (human’s perspective on risk), a risk was defined as the result of threat vs. disapproval through a relationship between the objective (threat) and the subjective (perception) [179]. The official agencies embraced this procedure for risk management [179,180]. Various studies highlighted that human perception on the processed food brings out higher risk awareness than natural food [181]. Alternatively, pesticides can also be considered as having risks for farmers, considering that pests can develop resistance against pesticides [182]. Consequently, a relationship was developed between the risk awareness of the pesticide risks to health and safety measures (for example protection equipment usage) [183].

Trust is hard to define or measure, and includes features from economy, psychology, and sociology. The most intensively used definition highlights trust as “*the willingness of a party to be vulnerable to the actions of another party based on the expectation that the other will perform a particular action important to the trustor, irrespective of the ability to monitor or control that other party*” [184]. To understand buyers’ trust in greengrocery, a detailed survey must be done [185]. People wanting to buy safe and quality grocery also seek the product’s origin [186]. Previous investigation has revealed that people’s trust in aliments is low, the potential explanation being the occurrence of various reported undesired events, epidemic episodes, or distance from the production site [187]. All of these aspects can weaken the trust in the food chain. Systematic media press releases on pesticide usage in various fruits and vegetables have suggested questionable ethics of farmers, highlighting once more that negative details regarding food safety can increase risk perspective. Consequently, buyers consider negative information more trustworthy than positive aspects [188]. The most reliable and valid instrument to evaluate trust through the food chain is the Trust in Food Toolkit [185]. The kit includes various parameters to evaluate consumers’ trust in greengrocery and the market chain, allowing indicators selection to estimate trust in accordance with the study aim [185]. The Trust in Food toolkit allows interpersonal trust or scepticism to be defined as personality features, as well as general trust [185]. General trust can be defined as a universal outlook or faculty to develop trust in other persons or things [189], therefore being less influenced by certain market incidents [187]. Instead, specific trust is associated with affective and cognitive features, for example brand loyalty [187]. Therefore, various studies considered specific trust as trust/distrust in the farm, trust/distrust in the greengrocery market chain, or trust/distrust in the fruits or vegetables [187]. It can be concluded that trust in the fruits or vegetables can predict the buyer’s intention, affecting market dynamics [187].

Various studies approached consumer concern about the health and environmental risks of pesticides. For example, the use of six common pesticides was analysed in terms of the impact of risk perspective on preferences [190]. It was observed that buyers considered the synthetic pesticide usage as the riskiest, the risk perspective directly influencing the outcomes [190]. In Italy, a high-risk perspective for both pesticide-related health and environmental consequences was highlighted [191]. Consequently, farmers try to implement as much as possible a pesticide-free production program, in accordance with the perception of environmental safety and human welfare [192].

When fruits or vegetables are chosen, risk perception features as a mediator, people taking their options and decisions based on tolerating high risks if there are some benefits, and vice versa [187]. It was observed that buying intention was notably determined by risk perspective when traceable food labels were taken into consideration and understood [193]. Consequently, risk perception could be a negative arbiter between food trust and acquisition intention. To rephrase, an increased risk perception on pesticides may explain how trust in a product influences the purchase intention, the relationship between these two parameters being bidirectional.

An important study was achieved in Brazil, concerning consumers’ trust and perception on conventionally produced vegetables and the regulation of pesticide usage, also taking into consideration risk perception and food price as potential indicators [187]. The conclusion was that people’s intention to buy conventionally produced vegetables was negatively forecasted by risk perception and positively prognosticated by trust in the product. Furthermore, risk perspective was found to moderate the influence of trust in the product on its acquisition [187].

A Greek investigation revealed that the most important positive parameter for influencing the acquisition of traditional vegetables was the recognized benefit of pesticide use to guarantee enough food, but also food security [194]. A general perception was observed: people handling and using pesticides, as well as buyers, may be protected from inherent risks if they adopt various actions, for example an adequate pesticide usage within farming activity [194]. However, the end-user may be prejudiced when applying home approaches to reduce pesticide residues (washing, peeling, or soaking), resulting in a small depletion of residual pesticides; meanwhile, other household practices, such as baking, boiling, canning, or juicing) prove to be ineffective [195].

## 7. Conclusions and Future Perspectives

The presence of pesticide residues or their metabolites within food must be of significant concern for worldwide consumers. Various procedures were approached to reduce or totally remove pesticides from agricultural products. The simplest and most convenient method is washing the fruits and vegetables, every consumer being able to thoroughly use it. Moreover, chlorine and other chemical agents proved to be efficient in pesticide level reduction. Nevertheless, the conventional approaches have disadvantages, like low efficiency, but also unwanted effects such as those that chemical cleansers have on human health. To overcome these disadvantages, emergent technologies (ozonification, ultrasonication, irradiation, cold plasma, or pulsed electric field) must be approached for the removal of pesticides from fruits and vegetables. One of the most efficient at lab scale proved to be cold plasma (a plasma which is not in thermodynamic equilibrium), resulting in the highest percentage of pesticide mitigation without affecting the food quality characteristics. Even though these procedures proved their efficacy in the removal of pesticides from fruits and vegetables, a huge gap still exists between research on a laboratory level and industrial application. Supplementary research efforts and scaling-up are required so that these novel procedures can be applied at an industrial level. Various intermediate products are formed during the degradation of pesticide residues, depending on the type of treatment and pesticide. Also, a thorough investigation must be conducted on the intermediary products that may result from pesticide degradation, and, more importantly on their toxic effects. Furthermore, the used procedures must be cost-effective, so that the farmers increase their profitability and decrease food price.

It was proven that trust, mainly in the greengrocery chain, plays an important part in customer decisions. Consequently, it is mandatory to provide public regulations for a strict control on the whole chain. Although the EU has a comprehensive regulatory framework, the milestones related to the surveillance of the implementation process are compulsory items to be considered with respect to food safety.

Database mining provides valuable insights into the regulatory status and risk classifications of pesticides and helps in the identification of regulatory gaps across geopolitical areas. The discrepancies in pesticide regulations between the EU and US emphasize the need for a consolidated and harmonised implementation of the UN GHS recommendations. The classification of pesticides as environmental threats highlights the importance of considering their impact on various ecosystems in risk assessment.

Also, it is mandatory to expand the number of analysed crops, and, also, to increase funding in analytical labs, logistics, and qualified employees for sampling. Public policy promoting organic greengrocery production must be taken into consideration to reduce pesticide usage and to target organic food production. The development of efficient risk evaluation and information sheets that can be easily understood and distributed by mass-media must be encouraged.

## Figures and Tables

**Table 1:** Pesticide classification considering class and activity.

Pesticide Class	Example of Pesticides	Activity	Refs.
Avicides	4-Aminopyridine Polybutene Nicarbazin	Kill birds Repellent Reproductive inhibitor	[13,14]
Acaricides	Dichlorodiphenyltrichloroethane Diazinon Carbaryl	Kill ticks and mites	[13,15]
Biopesticides	*Bacillus thuringiensis*	Wide action	[16]
Defoliants	2,4-Dichlorophenoxyacetic acid 2,4,5-Trichlorophenoxyacetic acid	Plant foliage removal	[17]
Fumigants	Aluminium Phosphide Magnesium Phosphide	Kill various organisms	[18]
Fungicides	Triazoles	Kill fungi	[19]
Herbicides	Atrazine Bentazon Clethodim Clethodim Glyphosate	Kill weeds and other unwanted plants	[20]
Insecticides	Carbamates (carbamyl, carbaryl methomyl, propoxur, and carbofuran) Neonicotinoids (imidacloprid, acetamiprid, dinotefuran, thiamethoxam, and clothianidin) Pyrethroids (allethrin, bifenthrin, cyfluthrin, lambda-cyhalothrin, cypermethrin, deltamethrin, permethrin, d-phenothrin, resmethrin, and tetramethrin)	Kill insects	[21]
Larvicides	*Bacillus thuringiensis subspecies israelensis* *Saccharopolyspora spinose* *Lysinibacillus sphaericus*	Inhibit larval growth	[22]
Plant growth regulators	Auxins, gibberellins and cytokinins Abscisic acid	Promoters or inhibitors of plant growth	[23]
Rodenticides	Chlorophacinone Chlorophacinone Warfarin Bromadiolone Difenacoum Difenacoum Cholecalciferol Cholecalciferol	Control mice and other rodents	[24]
Silvicides	Cacodylic acid	Woody vegetation controller	[13]
Virucides	Scytovirin	Act against viruses	[25]

**Table 2:** Pesticide restrictions and maximum residue limits (MRLs) in common fruits and vegetables in Europe [32,34] and US [33,35,36].

Examples of Individual Products to Which the MRLs Apply	Apples (mg/kg)	Table Grapes (mg/kg)	Wine Grapes (mg/kg)	Potatoes (mg/kg)	Tomatoes (mg/kg)
Carbaryl (EC) *	0.01	0.01	0.01	0.01	0.01
Carbaryl (FDA) ^a^	12	10	10	2	5
Carbofuran (EC) *	0.001	0.002	0.002	0.001	0.002
Carbofuran (FDA) ***	N/A	N/A	N/A	N/A	N/A
Propiconazole (EC)	0.01	0.01	0.01	0.01	0.01
Propiconazole (FDA) ****	N/A	N/A	N/A	N/A	3
Propoxur (EC)	0.005	0.005	0.005	0.005	0.005
Propoxur (FDA) ^a^	N/A	N/A	N/A	N/A	N/A
Aldrin and Dieldrin (aldrin and dieldrin combined expressed as dieldrin) (EC) *	0.01	0.01	0.01	0.010	0.01
Aldrin (FDA) ^a^	0.03	0.05	0.05	0.1	0.05
Dieldrin (FDA) ^a^	0.03	0.05	0.05	0.1	0.05
DDT (EC) *	0.05	0.05	0.05	0.05	0.05
DDT (FDA) ***	0.1	0.05	0.05	1	0.05
Captan (EC)	10	0.03	0.02	0.03	1
Captan (FDA) ****	25	25	25	0.05	0.05
Lindane (gamma isomer of hexachlorocyclohexane (HCH)) (EC) **	0.01	0.01	0.01	0.01	0.01
Lindane (gamma isomer of hexachlorocyclohexane (HCH)) (FDA) ***	N/A	0.5	0.5	0.5	N/A
Endosulfan (sum of alpha and beta isomers and endosulfan-sulphate expressed as endosulfan) (EC) **	0.05	0.05	0.05	0.05	0.05
Endosulfan (FDA) ***	N/A	N/A	N/A	N/A	N/A
Chlordane (sum of cis- and trans-chlordane) (EC) *	0.01	0.01	0.01	0.01	0.01
Chlordane (sum of cis- and trans-chlordane) (FDA) ***	0.1	0.1	0.1	0.1	0.1
Diazinon (EC) **	0.01	0.01	0.01	0.01	0.01
Diazinon (FDA) ****	0.5	N/A	N/A	0.1	0.75
Glyphosate (EC)	0.1	0.5	0.5	0.5	0.1
Glyphosate (FDA) ****	0.2	0.2	0.2	0.2	0.1
Malathion (EC) **	0.02	0.02	0.02	0.02	0.02
Malathion (FDA) ****	8	8	8	8	8
Parathion (EC) *	0.05	0.05	0.05	0.05	0.05
Parathion (FDA) ***	N/A	N/A	N/A	N/A	N/A
Parathion-methyl (sum of Parathion-methyl and paraoxon-methyl expressed as Parathion-methyl) (EC) *	0.01	0.01	0.01	0.01	0.01
Parathion-methyl (FDA) ***	N/A	N/A	N/A	N/A	N/A
Cypermethrin (cypermethrin including other mixtures of constituent isomers (sum of isomers)) (EC)	1	0.5	0.5	0.05	0.5
Cypermethrin (FDA) ***	N/A	N/A	N/A	N/A	N/A
Deltamethrin (cis-deltamethrin) (EC)	0.2	0.2	0.2	0.3	0.07
Deltamethrin (FDA) ****	0.2	N/A	N/A	0.04	0.2
Permethrin (EC) **	0.05	0.05	0.05	0.05	0.05
Permethrin (FDA) ****	0.05	2	2	0.05	2

* Banned or severely restricted in the EU. ** Banned as a plant protection product/severe restriction for other uses in the EU. *** 40 CFR 152.175: Pesticides classified for restricted use in US. **** Banned or restricted use in the US. a. Approved as a pesticide’s active ingredient for the mentioned fruits and vegetables in the US. N/A = not assigned.

**Table 3:** Solvents used for pesticide extraction from fruits and vegetables.

Fruits or Vegetables Type	Pesticides	Solvent	Refs.
Apples and tomatoes	Organophosphorus pesticides	Acetone	[94]
Fresh fruits and vegetables	90 different pesticides	Acetone	[95]
Lettuce, grapes, and tomatoes	Pesticide residue	Acetone	[96]
Fruits and vegetables	Organochlorine pesticides	Acetone, hexane	[97]
Apples, grapefruits, oranges, peaches, and pears Cabbage, leek, lettuce, onion, potatoes, and tomatoes	Pesticide residues	Acetone, cyclohexane/ethyl acetate	[98]
Carrots and oranges	Organochlorine and organophosphorus pesticides Fungicides	Acetonitrile	[99]
Oranges	Pesticides residues	Methanol	[100]
Beetroot	Cyromazine and its transformation products	Methanol	[101]
Oranges	Pesticides residues	Methanol, ethyl acetate	[102]
Broccoli, cauliflower, green pepper, komatsuna, okra, and spinach	Pesticides residues	Ethyl acetate	[103]
Oranges	Fungicides and insecticides	Ethyl acetate	[100]

**Table 4:** Examples of pesticide determination in fruits and vegetables.

Fruits/Vegetables Type	Pesticide Type	Determination Method	Limit of Detection (µg/kg)	Limit of Quantification (mg/kg)	Refs.
Apples, apple juice, and tomatoes	Dichlorvos, diazinon, fenitrothion, malathion, and parathion	GC-FPD	3 × 10^−3^–9 × 10^−3^	-	[94]
Apples, oranges, and pears	Acephate, fenitrothion, methamidophos, and omethoate	GC-MS	1 × 10^−2^	1 × 10^−2^	[95]
Carrots and oranges	Acephate, aldrin, chlorpyrifos, diazinon, dichlorvos, dieldrin, fenthion, methamidophos, methoxychlor, methyl parathion, and others	GC-MS	1 × 10^−1^	4 × 10^−4^–9 × 10^−2^	[99]
Oranges	Carbendazim, fenthion, imidacloprid, methidathion, methiocarb, trichlorfon, and others	LC-MS	1 × 10^0^–5 × 10^1^	1 × 10^−3^–5 × 10^−2^	[100]
Beetroot	Cyromazine and melamine	LC-ESI-MS	1 × 10^1^	5 × 10^−1^	[101]
Herbs	Parathion, phosmet, phosphamidone, propoxur, simazine, terbuthylazine, terrazole, and others	GC-MS	6.2 × 10^1^–12×10^1^	5 × 10^−1^	[111]
Cherries and strawberries	Azinphos-methyl, malathion, methidathion, and others	GC-MS	1 × 10^0^	5 × 10^−2^–5 × 10^−1^	[112]
Oranges, pears, and tomatoes	Azinphos-methyl, malathion, methidathion, and others	LC-MS	1 × 10^1^–1 × 10^2^	5 × 10^−3^–5 × 10^−1^	[113]

GC-FPD—gas chromatography-flame photometric detector; GC-MS—gas chromatography-mass spectrometry; LC-MS—liquid chromatography-mass spectrometry; LC-ESI-MS-liquid chromatography-electrospray ionization-tandem mass spectrometry.

## Data Availability

Not applicable.

## Supplementary Materials

The following supporting information can be downloaded at: https://www.mdpi.com/article/10.3390/jox14040088/s1, Table S1. Examples of food processing techniques to mitigate the residual pesticides, Table S2: GHS Classification of investigated pesticides.
