# Supplementary material for: Consumer Safety and Pesticide Residues: Evaluating Mitigation Protocols for Greengrocery
Source: J Xenobiot. 2024 Nov 1;14(4):1638–69. doi: 10.3390/jox14040088 (PMC11587004; doi:10.3390/jox14040088)
Supplement: Supplementary file 1 [file jox-14-00088-s001.zip › jox-3068639-supplementary.pdf]

Supplementary Material:
Consumer Safety and Pesticide Residues: Evaluating Mitigation Protocols for Greengrocery

Diana Ionela Popescu (Stegarus) [1], Corina Mihaela Oprita (Cioara) [2], Radu Tamaian [1] and Violeta-Carolina Niculescu [1,*]

[1] National Research and Development Institute for Cryogenic and Isotopic Technologies—ICSI Ramnicu Valcea, 4th Uzinei Street, P.O. Box Raureni 7, 240050 Ramnicu Valcea, Romania;
diana.stegarus@icsi.ro (D.I.P.); radu.tamaian@icsi.ro (R.T.)
[2] Doctoral School of Applied Sciences, Ovidius University Constanta, 124 Mamaia Blvd, 1st University Alley, 900470 Constanta, Romania; cioara.corina-ct@ansvsa.ro
* Correspondence: violeta.niculescu@icsi.ro

Table S1. Examples of food processing techniques to mitigate the residual pesticides

Fruits or vegetables type	Experimental assay	Removal tests	Ref.
peaches (cv. Spring Flame)<br>nectarines (cv. Nectafun)<br>andapricots (cv. PinkCot)	The fruits were immersed for 10 min in the pesticide's solution, followed by drying for 1 h. Then, they were completely submerged for 5, 15 and 25 min in the treatment solution. Dozes of 5 g/L, 2mL/L, 0.7 mL/L were prepared for cyprodinil, iprodione and tebuconazole	Washing:<br>- tap water;<br>- water acidified with 80 mg/L citric acid and 1 g/L NaCl; 1 h operation under a coupled refrigerating system (0–2 °C);<br>- water with ClO_2 of 10 and 20 mg/L (15 min) folowed by phtococatalysis	[132]
green pepper (*Capsicum annuum* L.) sweet var. California Wonder, hot pepper var. Long Red Cayonne and eggplant (*Solanum melongena* L.) var. Balady	Plants were sprayed with profenofos once at the recommended rate (750 mL profenofos /0.42 ha), 2 days before the first harvesting. Fruit samples were randomly collected (500 g/replicate) at intervals of 1 h), 1, 3, 5, 7, 10 and 14 days after application	- carried out on the 10th and 7th day treated pepper and eggplant fruits:<br>- rinsing with tap water, soap 1%, $KMnO_4$ 0.01%, sodium chloride 1%, NaOH 0.1% and CH_3COOH 2% for 1 min;<br>- blanching: the fruit was boiled in water for 5 min and allowed to dry on a clean paper;<br>- frying: the fruit was fried in oil for 5 min and dried<br>- pickling: the fruit was pickled in 10.5% NaCl solution for 1-2 weeks	[140]

apples	30 apple trees were treated with pesticide, using an air blast sprayer (Captan - 3.75 kg/ha)	- no preparation;<br>- rinse with de-ionized water for 10-15 s and continuous rubbing by hand;<br>- rinse and peeling with a paring knife	[141]
tomatoes	cherry tomatoes (1200 g) were contaminated with a mixture of nine pesticides - acephate, malathion, carbaryl, bifenthrin, cypermethrin, cyhalothrin, permethrin, chlorothalonil, and imidacloprid (120 μg/mL); time of contact: 5 s	washing:<br>- 55 g tomatoes were washed with 275 mL of water, NaOCl (80 μg/mL), peroxyacetic acid (80 μg/mL) ot Tween 0 (0.1%); T=10 °C; 600 rpm; t=1 min<br>- washing with and without sonication	[142]
raw cucumber	Fresh cucumbers were collected with no previous pesticide application; 5 g cucumber were immersed in pesticides solution for 50 min (trichlorfon, dimethoate, dichlorvos, fenitrothion, and chlorpyrifos)	- washing: cucumbers were soaked in NaCl, CH_3COOH, $NaHCO_3$ or Na_2COO_3 for 5, 10 and 20 min;<br>-ultrasonication: spiked samples was soaked in water and ultrasonicated for 5, 10 and 20 min	[143]
wheat grains	Wheat grains were collected from vertical silos, cleaned and dried up to 60 °C, then spiked with 100 μL of fenitrothion and deltamethrin solutions (1000 μg/mL) under stirring (2 min).	- 50 spiked grains were transffered at the top part of a silo on a polyamide screen surface; ozon gas was applied (60 μmol/mol) for 30-180 min	[145]
carrots	Pesticides were applied 80 days after planting (BBCH 49) in doses equivalent to five times the recommended doozes (3 L/ha 25% difenoconazole and 11 L/ha 45% linuron)	- O_3 as gas (2 L/min) in a 0.075 m3 acrylic chamber<br>- O_3 dissolved in waterin a circular chamber of PVC (50 × 80 cm)	[147]
strawberry	10 g fresh strawberries (from maket) were fortified with carbendazim and azoxystrobin (final dose of 6 mg/kg)	- irradiation with Gamma Cell 220 cobalt-60 (60 Co) gamma-ray source loaded with 2638.18 Ci, (ambiental emperature, dose rate of 0.16 kGy/h)	[151]
eggplants, capsicum, cucumbers and tomatoes	Vegetables were monitored to determine carbaryl, carbofuran, pirimicarb, phenthoate, diazinon, parathion, dimethoate, phosphamidon, and pirimiphos-methyl; they were washed with tap water, stems were removed; cucumbers were peeled	- radiation: two different radiation doses (0.5 and 1.0 kGy) were applied; a 1850 terabecquerel (50 kCi) 60Co gamma-irradiator was used as the radiation source.	[152]

	and sliced, capsicum was chped and tomatoes were sliced without peeling		
blueberry	Fresh blueberries were purchased from local supermarket and degradation of boscalid and imidacloprid was monitored by placing the samples in a PET package within the inter-dielectric space	- cold plasma exposure: an in-package high voltage dielectric barrier discharge plasma reactor was used (time: 0, 2, 5 min and voltage of 60 and 80 kV; ambiental temperature)	[156]
tomatoes	Uniformly sized tomatoes (Marutham) were purchased from a local market; the washed samples were immerded in chlorpyrifos solution (5 min) with various concentrations: 0.6, 0.7, and 0.8 mg/L	- low-pressure nonthermal dielectric barrier discharge plasma exposure: pressure 50–75 mm Hg; two aluminum electrodes coated with 2 mm glass dielectric; plasma of powers 2.0, 3.0, and 5.0 W; exposure time of 4–6 min	[157]
mango	Mangoes (90–100 days after full bloom) were purchased from market to monitore removal of chlorpyrifos and cypermethrin; pesticide solution (100 ppm) was sprayed ono mango fruits (10 mL/fruit) using glass sprayers under a fume hood	- non-thermal plasma exposure: a gliding arc (GA) discharge was used for 5 and 10 min; mango fruit was put in a glass beaker with 1 L distilled water, then Ar gas and micro-bubble water were passed through GA discharge and transferred in the beaker (temperature 28–30 °C; Ar flow rate 2-8 L/min)	[158]
goji berry	Goji berries were fortified with omethoate and dichlorvos (5 mg/l) by spraying	- gas phase surface discharge plasma: samples were exposed for 0-30 min at voltage of 5, 8, 10, 12, 15, and 20 kV.	[159]
apples	Source of apples was not provided; surface of fresh apples was sprayed with paraoxon solution (10%v/v methanol) at 0-100,000 ppb	- atmospheric air plasma exposure: samples were exposed to the plasma in a refrigerator (plasma flow rate 4 L/min; time 10-15 min; voltage 4 kV; air purity 99.9%)	[160]

Table S2. GHS Classification of investigated pesticides

Compound	CAS Registry Number	GHS Hazard Statements	
		By EPA*	By ECHA**
**Carbaryl**	63-25-2	H301 (64.42%): Toxic if swallowed [Danger: Acute toxicity, oral]<br>H302 (35.58%): Harmful if swallowed [Warning: Acute toxicity, oral]<br>H332 (97.12%): Harmful if inhaled [Warning: Acute toxicity, inhalation]<br>H351 (100%): Suspected of causing cancer [Warning: Carcinogenicity]<br>H400 (100%): Very toxic to aquatic life [Warning: Hazardous to the aquatic environment, acute hazard]<br>H410 (70.19%): Very toxic to aquatic life with long lasting effects [Warning: Hazardous to the aquatic environment, long-term hazard]	H302: Harmful if swallowed [Warning: Acute toxicity, oral]<br>H332: Harmful if inhaled [Warning: Acute toxicity, inhalation]<br>H351: Suspected of causing cancer [Warning: Carcinogenicity]<br>H400: Very toxic to aquatic life [Warning: Hazardous to the aquatic environment, acute hazard]
**Carbofuran**	1563-66-2	H300+H330 (21.88%): Fatal if swallowed or if inhaled [Danger: Acute toxicity, oral; acute toxicity, inhalation]<br>H300 (100%): Fatal if swallowed [Danger: Acute toxicity, oral]<br>H330 (100%): Fatal if inhaled [Danger: Acute toxicity, inhalation]<br>H400 (100%): Very toxic to aquatic life [Warning: Hazardous to the aquatic environment, acute hazard]<br>H410 (100%): Very toxic to aquatic life with long lasting effects [Warning: Hazardous to the aquatic environment, long-term hazard]	H300: Fatal if swallowed [Danger: Acute toxicity, oral]<br>H330: Fatal if inhaled [Danger: Acute toxicity, inhalation]<br>H410: Very toxic to aquatic life with long lasting effects [Warning: Hazardous to the aquatic environment, long-term hazard]
**Propiconazole**	60207-90-1	H302 (100%): Harmful if swallowed [Warning: Acute toxicity, oral]<br>H317 (100%): May cause an allergic skin reaction [Warning: Sensitization, Skin]	H302: Harmful if swallowed [Warning: Acute toxicity, oral]<br>H317: May cause an allergic skin reaction [Warning: Sensitization, Skin]

		H360 (13.45%): May damage fertility or the unborn child [Danger: Reproductive toxicity]<br>H360D (35.67%): May damage the unborn child [Danger: Reproductive toxicity]<br>H400 (100%): Very toxic to aquatic life [Warning: Hazardous to the aquatic environment, acute hazard]<br>H410 (100%): Very toxic to aquatic life with long lasting effects [Warning: Hazardous to the aquatic environment, long-term hazard]	H360D: May damage the unborn child [Danger: Reproductive toxicity]<br>H410: Very toxic to aquatic life with long lasting effects [Warning: Hazardous to the aquatic environment, long-term hazard]
**Propoxur**	114-26-1	H300 (23.31%): Fatal if swallowed [Danger: Acute toxicity, oral]<br>H301 (44.79%): Toxic if swallowed [Danger: Acute toxicity, oral]<br>H311 (32.52%): Toxic in contact with skin [Danger: Acute toxicity, dermal]<br>H331 (22.7%): Toxic if inhaled [Danger: Acute toxicity, inhalation]<br>H400 (100%): Very toxic to aquatic life [Warning: Hazardous to the aquatic environment, acute hazard]<br>H410 (68.1%): Very toxic to aquatic life with long lasting effects [Warning: Hazardous to the aquatic environment, long-term hazard]	H301: Toxic if swallowed [Danger: Acute toxicity, oral]<br>H410: Very toxic to aquatic life with long lasting effects [Warning: Hazardous to the aquatic environment, long-term hazard]
**Aldrin**	309-00-2	H300 (60%): Fatal if swallowed [Danger: Acute toxicity, oral]<br>H301 (40%): Toxic if swallowed [Danger: Acute toxicity, oral]<br>H310 (70%): Fatal in contact with skin [Danger: Acute toxicity, dermal]<br>H311 (30%): Toxic in contact with skin [Danger: Acute toxicity, dermal]<br>H351 (100%): Suspected of causing cancer [Warning: Carcinogenicity]<br>H372 (100%): Causes damage to organs through prolonged or repeated exposure	H301: Toxic if swallowed [Danger: Acute toxicity, oral]<br>H311: Toxic in contact with skin [Danger: Acute toxicity, dermal]<br>H351: Suspected of causing cancer [Warning: Carcinogenicity]<br>H372: Causes damage to organs through prolonged or repeated exposure [Danger: Specific target organ toxicity, repeated exposure]<br>H410: Very toxic to aquatic life with long lasting effects [Warning: Hazardous to the aquatic environment, long-term hazard]

		[Danger: Specific target organ toxicity, repeated exposure]<br>H400 (100%): Very toxic to aquatic life [Warning: Hazardous to the aquatic environment, acute hazard]<br>H410 (100%): Very toxic to aquatic life with long lasting effects [Warning: Hazardous to the aquatic environment, long-term hazard]	
**Dieldrin**	60-57-1	H300 (15.79%): Fatal if swallowed [Danger: Acute toxicity, oral]<br>H301 (84.21%): Toxic if swallowed [Danger: Acute toxicity, oral]<br>H310 (100%): Fatal in contact with skin [Danger: Acute toxicity, dermal]<br>H330 (15.79%): Fatal if inhaled [Danger: Acute toxicity, inhalation]<br>H351 (100%): Suspected of causing cancer [Warning: Carcinogenicity]<br>H372 (100%): Causes damage to organs through prolonged or repeated exposure [Danger: Specific target organ toxicity, repeated exposure]<br>H400 (100%): Very toxic to aquatic life [Warning: Hazardous to the aquatic environment, acute hazard]<br>H410 (100%): Very toxic to aquatic life with long lasting effects [Warning: Hazardous to the aquatic environment, long-term hazard]	H301: Toxic if swallowed [Danger: Acute toxicity, oral]<br>H310: Fatal in contact with skin [Danger: Acute toxicity, dermal]<br>H351: Suspected of causing cancer [Warning: Carcinogenicity]<br>H372: Causes damage to organs through prolonged or repeated exposure [Danger: Specific target organ toxicity, repeated exposure]<br>H410: Very toxic to aquatic life with long lasting effects [Warning: Hazardous to the aquatic environment, long-term hazard]
**DDT**	50-29-3	H301+H311 (43.68%): Toxic if swallowed or in contact with skin [Danger: Acute toxicity, oral; acute toxicity, dermal]<br>H301 (98.85%): Toxic if swallowed [Danger: Acute toxicity, oral]<br>H311 (50.57%): Toxic in contact with skin [Danger: Acute toxicity, dermal]<br>H351 (98.85%): Suspected of causing cancer [Warning: Carcinogenicity]	H301: Toxic if swallowed [Danger: Acute toxicity, oral]<br>H351: Suspected of causing cancer [Warning: Carcinogenicity]<br>H372: Causes damage to organs through prolonged or repeated exposure [Danger: Specific target organ toxicity, repeated exposure]

		H372 (100%): Causes damage to organs through prolonged or repeated exposure [Danger: Specific target organ toxicity, repeated exposure]<br>H400 (100%): Very toxic to aquatic life [Warning: Hazardous to the aquatic environment, acute hazard]<br>H410 (98.85%): Very toxic to aquatic life with long lasting effects [Warning: Hazardous to the aquatic environment, long-term hazard]	H410: Very toxic to aquatic life with long lasting effects [Warning: Hazardous to the aquatic environment, long-term hazard]
**Captan**	133-06-2	H317 (100%): May cause an allergic skin reaction [Warning: Sensitization, Skin]<br>H318 (100%): Causes serious eye damage [Danger: Serious eye damage/eye irritation]<br>H331 (100%): Toxic if inhaled [Danger: Acute toxicity, inhalation]<br>H351 (100%): Suspected of causing cancer [Warning: Carcinogenicity]<br>H400 (100%): Very toxic to aquatic life [Warning: Hazardous to the aquatic environment, acute hazard]	H317: May cause an allergic skin reaction [Warning: Sensitization, Skin]<br>H318: Causes serious eye damage [Danger: Serious eye damage/eye irritation]<br>H331: Toxic if inhaled [Danger: Acute toxicity, inhalation]<br>H340: May cause genetic defects [Danger: Germ cell mutagenicity]<br>H351: Suspected of causing cancer [Warning: Carcinogenicity]<br>H400: Very toxic to aquatic life [Warning: Hazardous to the aquatic environment, acute hazard]
**Lindane**<br>**(γ -HCH)**	58-89-9***	BANNED;<br>GHS labelling is no more available	H301: Toxic if swallowed [Danger: Acute toxicity, oral]<br>H312: Harmful in contact with skin [Warning: Acute toxicity, dermal]<br>H332: Harmful if inhaled [Warning:: Acute toxicity, inhalation]<br>H362: May cause harm to breast-fed children [Warning: Reproductive toxicity, effects on or via lactation]<br>H373: May causes damage to organs through prolonged or repeated exposure [Warning: Specific target organ toxicity, repeated exposure]

			H400: Very toxic to aquatic life [Warning: Hazardous to the aquatic environment, acute hazard]<br>H410: Very toxic to aquatic life with long lasting effects [Warning: Hazardous to the aquatic environment, long-term hazard]
**Endosulfan**	115-29-7	H300+H330 (32.94%): Fatal if swallowed or if inhaled [Danger: Acute toxicity, oral; acute toxicity, inhalation]<br>H300 (96.47%): Fatal if swallowed [Danger: Acute toxicity, oral]<br>H312 (96.47%): Harmful in contact with skin [Warning: Acute toxicity, dermal]<br>H330 (96.47%): Fatal if inhaled [Danger: Acute toxicity, inhalation]<br>H400 (100%): Very toxic to aquatic life [Warning: Hazardous to the aquatic environment, acute hazard]<br>H410 (100%): Very toxic to aquatic life with long lasting effects [Warning: Hazardous to the aquatic environment, long-term hazard]	H300: Fatal if swallowed [Danger: Acute toxicity, oral]<br>H312: Harmful in contact with skin [Warning: Acute toxicity, dermal]<br>H330: Fatal if inhaled [Danger: Acute toxicity, inhalation]<br>H410: Very toxic to aquatic life with long lasting effects [Warning: Hazardous to the aquatic environment, long-term hazard]
**Chlordane** ****	57-74-9	H302 (100%): Harmful if swallowed [Warning: Acute toxicity, oral]<br>H351 (86.96%): Suspected of causing cancer [Warning: Carcinogenicity]<br>H400 (95.65%): Very toxic to aquatic life [Warning: Hazardous to the aquatic environment, acute hazard]	H302+H332 (82.61%): Harmful if swallowed or if inhaled [Warning: Acute toxicity, oral; acute toxicity, inhalation]<br>H302 (95.65%): Harmful if swallowed [Warning: Acute toxicity, oral]<br>H311 (86.96%): Toxic in contact with skin [Danger: Acute toxicity, dermal]<br>H315 (86.96%): Causes skin irritation [Warning: Skin corrosion/irritation]<br>H319 (86.96%): Causes serious eye irritation [Warning: Serious eye damage/eye irritation]<br>H332 (86.96%): Harmful if inhaled [Warning: Acute toxicity, inhalation]<br>H335 (100%): May cause respiratory irritation [Warning: Specific target organ toxicity, single exposure; Respiratory tract irritation]

			H400 (95.65%): Very toxic to aquatic life [Warning: Hazardous to the aquatic environment, acute hazard]<br>H410 (82.61%): Very toxic to aquatic life with long lasting effects [Warning: Hazardous to the aquatic environment, long-term hazard]
**Diazinon**	333-41-5	H302 (73.15%): Harmful if swallowed [Warning: Acute toxicity, oral]<br>H312 (24.54%): Harmful in contact with skin [Warning: Acute toxicity, dermal]<br>H341 (16.2%): Suspected of causing genetic defects [Warning: Germ cell mutagenicity]<br>H350 (16.67%): May cause cancer [Danger: Carcinogenicity]<br>H370 (16.67%): Causes damage to organs [Danger: Specific target organ toxicity, single exposure]<br>H373 (16.67%): May causes damage to organs through prolonged or repeated exposure [Warning: Specific target organ toxicity, repeated exposure]<br>H400 (99.54%): Very toxic to aquatic life [Warning: Hazardous to the aquatic environment, acute hazard]<br>H410 (75.93%): Very toxic to aquatic life with long lasting effects [Warning: Hazardous to the aquatic environment, long-term hazard]	H302: Harmful if swallowed [Warning: Acute toxicity, oral]<br>H410: Very toxic to aquatic life with long lasting effects [Warning: Hazardous to the aquatic environment, long-term hazard]
**Glyphosate**	1071-83-6	H302 (100%): Harmful if swallowed [Warning: Acute toxicity, oral]<br>H411 (100%): Toxic to aquatic life with long lasting effects [Hazardous to the aquatic environment, long-term hazard]	H318 (99.64%): Causes serious eye damage [Danger: Serious eye damage/eye irritation]<br>H411 (99.64%): Toxic to aquatic life with long lasting effects [Hazardous to the aquatic environment, long-term hazard]
**Malathion**	121-75-5	H302 (97.2%): Harmful if swallowed [Warning: Acute toxicity, oral]<br>H317 (98.13%): May cause an allergic skin reaction [Warning: Sensitization, Skin]	H302: Harmful if swallowed [Warning: Acute toxicity, oral]<br>H317: May cause an allergic skin reaction [Warning: Sensitization, Skin]

		H331 (17.29%): Toxic if inhaled [Danger: Acute toxicity, inhalation]<br>H400 (100%): Very toxic to aquatic life [Warning: Hazardous to the aquatic environment, acute hazard]<br>H410 (100%): Very toxic to aquatic life with long lasting effects [Warning: Hazardous to the aquatic environment, long-term hazard]	H410: Very toxic to aquatic life with long lasting effects [Warning: Hazardous to the aquatic environment, long-term hazard]
**Parathion**	56-38-2	H300 (100%): Fatal if swallowed [Danger: Acute toxicity, oral]<br>H310 (12.77%): Fatal in contact with skin [Danger: Acute toxicity, dermal]<br>H311 (87.23%): Toxic in contact with skin [Danger: Acute toxicity, dermal]<br>H330 (100%): Fatal if inhaled [Danger: Acute toxicity, inhalation]<br>H372 (100%): Causes damage to organs through prolonged or repeated exposure [Danger: Specific target organ toxicity, repeated exposure]<br>H400 (100%): Very toxic to aquatic life [Warning: Hazardous to the aquatic environment, acute hazard]<br>H410 (100%): Very toxic to aquatic life with long lasting effects [Warning: Hazardous to the aquatic environment, long-term hazard]	H300: Fatal if swallowed [Danger: Acute toxicity, oral]<br>H311: Toxic in contact with skin [Danger: Acute toxicity, dermal]<br>H330: Fatal if inhaled [Danger: Acute toxicity, inhalation]<br>H372: Causes damage to organs through prolonged or repeated exposure [Danger: Specific target organ toxicity, repeated exposure]<br>H410: Very toxic to aquatic life with long lasting effects [Warning: Hazardous to the aquatic environment, long-term hazard]
**Parathion-methyl**	298-00-0	H226 (87.5%): Flammable liquid and vapor [Warning: Flammable liquids]<br>H300 (100%): Fatal if swallowed [Danger: Acute toxicity, oral]<br>H311 (87.5%): Toxic in contact with skin [Danger: Acute toxicity, dermal]<br>H330 (100%): Fatal if inhaled [Danger: Acute toxicity, inhalation]<br>H373 (100%): May causes damage to organs through prolonged or repeated exposure [Warning: Specific target organ toxicity, repeated exposure]	H226: Flammable liquid and vapor [Warning: Flammable liquids]<br>H300: Fatal if swallowed [Danger: Acute toxicity, oral]<br>H311: Toxic in contact with skin [Danger: Acute toxicity, dermal]<br>H330: Fatal if inhaled [Danger: Acute toxicity, inhalation]<br>H373: May causes damage to organs through prolonged or repeated exposure [Warning: Specific target organ toxicity, repeated exposure]

		H400 (87.5%): Very toxic to aquatic life [Warning: Hazardous to the aquatic environment, acute hazard]<br>H410 (87.5%): Very toxic to aquatic life with long lasting effects [Warning: Hazardous to the aquatic environment, long-term hazard]	H410: Very toxic to aquatic life with long lasting effects [Warning: Hazardous to the aquatic environment, long-term hazard]
**Cypermethrin**	52315-07-8	H301 (99.54%): Toxic if swallowed [Danger: Acute toxicity, oral]<br>H332 (41.67%): Harmful if inhaled [Warning: Acute toxicity, inhalation]<br>H335 (100%): May cause respiratory irritation [Warning: Specific target organ toxicity, single exposure; Respiratory tract irritation]<br>H373 (100%): May causes damage to organs through prolonged or repeated exposure [Warning: Specific target organ toxicity, repeated exposure]<br>H400 (99.54%): Very toxic to aquatic life [Warning: Hazardous to the aquatic environment, acute hazard]<br>H410 (100%): Very toxic to aquatic life with long lasting effects [Warning: Hazardous to the aquatic environment, long-term hazard]	H301 (25.9%): Toxic if swallowed [Danger: Acute toxicity, oral]<br>H302+H332 (10.21%): Harmful if swallowed or if inhaled [Warning: Acute toxicity, oral; acute toxicity, inhalation]<br>H302 (74.1%): Harmful if swallowed [Warning: Acute toxicity, oral]<br>H332 (67.86%): Harmful if inhaled [Warning: Acute toxicity, inhalation]<br>H335 (100%): May cause respiratory irritation [Warning: Specific target organ toxicity, single exposure; Respiratory tract irritation]<br>H373 (31.95%): May causes damage to organs through prolonged or repeated exposure [Warning: Specific target organ toxicity, repeated exposure]<br>H400 (88.66%): Very toxic to aquatic life [Warning: Hazardous to the aquatic environment, acute hazard]<br>H410 (100%): Very toxic to aquatic life with long lasting effects [Warning: Hazardous to the aquatic environment, long-term hazard]
**Deltamethrin**	52918-63-5	H300 (28.01%): Fatal if swallowed [Danger: Acute toxicity, oral]<br>H301 (71.99%): Toxic if swallowed [Danger: Acute toxicity, oral]<br>H317 (10.84%): May cause an allergic skin reaction [Warning: Sensitization, Skin]<br>H319 (10.84%): Causes serious eye irritation [Warning: Serious eye damage/eye irritation]	H301: Toxic if swallowed [Danger: Acute toxicity, oral]<br>H331: Toxic if inhaled [Danger: Acute toxicity, inhalation]<br>H410: Very toxic to aquatic life with long lasting effects [Warning: Hazardous to the aquatic environment, long-term hazard]

		H331 (100%): Toxic if inhaled [Danger: Acute toxicity, inhalation]<br>H335 (10.84%): May cause respiratory irritation [Warning: Specific target organ toxicity, single exposure; Respiratory tract irritation]<br>H361 (10.84%): Suspected of damaging fertility or the unborn child [Warning: Reproductive toxicity]<br>H372 (10.84%): Causes damage to organs through prolonged or repeated exposure [Danger: Specific target organ toxicity, repeated exposure]<br>H400 (99.4%): Very toxic to aquatic life [Warning: Hazardous to the aquatic environment, acute hazard]<br>H410 (100%): Very toxic to aquatic life with long lasting effects [Warning: Hazardous to the aquatic environment, long-term hazard]	
**Permethrin**	52645-53-1	H302 (100%): Harmful if swallowed [Warning: Acute toxicity, oral]<br>H317 (88.85%): May cause an allergic skin reaction [Warning: Sensitization, Skin]<br>H332 (100%): Harmful if inhaled [Warning: Acute toxicity, inhalation]<br>H400 (99.81%): Very toxic to aquatic life [Warning: Hazardous to the aquatic environment, acute hazard]<br>H410 (100%): Very toxic to aquatic life with long lasting effects [Warning: Hazardous to the aquatic environment, long-term hazard]	H302: Harmful if swallowed [Warning: Acute toxicity, oral]<br>H371: May cause damage to organs [Warning: Specific target organ toxicity, single exposure]

* EPA (U.S. Environmental Protection Agency) use a standard GHS (Globally Harmonized System) classification; ** ECHA (European Chemicals Agency) use a harmonised GHS classification and labelling approved by the European Union – CLP (Classification, Labelling and Packaging) Regulation (EC) No 1272/2008; *** same CAS Registry Number for all isomers of HCH; **** technical chlordane is not a single chemical compound – it is a mixture of chlordane (*cis*-chlordane and *trans*-chlordane) with a series of related chemical compounds).
